# A general modeling framework for describing spatially structured population dynamics

**DOI:** 10.1002/ece3.3685

**Published:** 2017-11-30

**Authors:** Christine Sample, John M. Fryxell, Joanna A. Bieri, Paula Federico, Julia E. Earl, Ruscena Wiederholt, Brady J. Mattsson, D. T. Tyler Flockhart, Sam Nicol, Jay E. Diffendorfer, Wayne E. Thogmartin, Richard A. Erickson, D. Ryan Norris

**Affiliations:** ^1^ Department of Mathematics Emmanuel College Boston MA USA; ^2^ Department of Integrative Biology University of Guelph Guelph ON Canada; ^3^ Department of Mathematics University of Redlands Redlands CA USA; ^4^ Department of Mathematics, Computer Science and Physics Capital University Columbus OH USA; ^5^ School of Biological Sciences Louisiana Tech University Ruston LA USA; ^6^ Everglades Foundation Palmetto Bay FL USA; ^7^ Institute of Silviculture University of Natural Resources and Life Sciences Vienna Austria; ^8^ CSIRO Land and Water, EcoSciences Precinct Dutton Park Qld Australia; ^9^ U.S. Geological Survey, Geosciences and Environmental Change Science Center Denver CO USA; ^10^ U.S. Geological Survey Upper Midwest Environmental Sciences Center La Crosse WI USA; ^11^ Present address: Institute of Wildlife Biology & Game Management University of Natural Resources & Life Sciences (BOKU) Vienna Austria

**Keywords:** connectivity, dispersal, metapopulations, migration, models, networks

## Abstract

Variation in movement across time and space fundamentally shapes the abundance and distribution of populations. Although a variety of approaches model structured population dynamics, they are limited to specific types of spatially structured populations and lack a unifying framework. Here, we propose a unified network‐based framework sufficiently novel in its flexibility to capture a wide variety of spatiotemporal processes including metapopulations and a range of migratory patterns. It can accommodate different kinds of age structures, forms of population growth, dispersal, nomadism and migration, and alternative life‐history strategies. Our objective was to link three general elements common to all spatially structured populations (space, time and movement) under a single mathematical framework. To do this, we adopt a network modeling approach. The spatial structure of a population is represented by a weighted and directed network. Each node and each edge has a set of attributes which vary through time. The dynamics of our network‐based population is modeled with discrete time steps. Using both theoretical and real‐world examples, we show how common elements recur across species with disparate movement strategies and how they can be combined under a unified mathematical framework. We illustrate how metapopulations, various migratory patterns, and nomadism can be represented with this modeling approach. We also apply our network‐based framework to four organisms spanning a wide range of life histories, movement patterns, and carrying capacities. General computer code to implement our framework is provided, which can be applied to almost any spatially structured population. This framework contributes to our theoretical understanding of population dynamics and has practical management applications, including understanding the impact of perturbations on population size, distribution, and movement patterns. By working within a common framework, there is less chance that comparative analyses are colored by model details rather than general principles.

## 1 INTRODUCTION

Understanding the processes shaping species distribution and abundance involves integrating three general elements that are characteristic of all populations: space, time, and movement (Brown, Mehlman, & Stevens, 1995; Gadgil, 1971; Leirs et al., 1997; MacArthur, 1972; Newton, 2010; Tilman & Kareiva, 1997). First, local conditions in space limit and regulate population growth (Pianka, 1970) and variable spatial conditions result in variation in species demographic rates and abundance (Tilman & Kareiva, 1997). Second, population abundance varies over time, whether expressed as seasonality (Leirs et al., 1997), stochastic variation (Brown et al., 1995), or time‐dependent variation in demographic processes (Newton, 2010). Third, given that most organisms are mobile to some degree, movement influences and interacts with spatiotemporal changes in local conditions (Kubisch, Holt, Poethke, & Fronhofer, 2014).

Mathematical models have contributed to understanding the processes driving spatiotemporal population dynamics (Brown, 1984; Collins & Glenn, 1991; Gaston & Lawton, 1990; Keeling, Wilson, & Pacala, 2000; Kerr, Neuhauser, Bohannan, & Dean, 2006; Kneitel & Miller, 2003). Mathematical approaches to simulate these processes include metapopulation models (Hanski & Hanski, 1999; Lamy, Gimenez, Pointier, Jarne, & David, 2013; Peterman et al. 2013), migratory network (Erickson, Thogmartin, Russell, Diffendorfer, & Szymanski, 2014; Mattsson et al., 2012; Taylor & Norris, 2010; Wiederholt et al., 2013), and dispersal (Rudnick et al., 2012; Kubisch et al. 2014) models. Each of these models has fundamentally different structure, so it can be challenging to synthesize results across spatial or temporal scales, or modify models to meet the specifications of different ecological systems.

Our objective was to link the three general elements common to all spatially structured populations (space, time, and movement) under a single mathematical framework that is flexible enough to capture a wide variety of spatiotemporal dynamics and movement strategies. To do this, we adopt a network modeling approach. Network models originated in the mathematical field of graph theory and have been subsequently adapted to a wide variety of biological fields such as disease dynamics, molecular biology, landscape ecology, and conservation biology (Minor & Urban, 2008; Proulx, Promislow, & Phillips, 2005; Urban, Minor, Treml, & Schick, 2009). With their flexible structure, network models have been successfully used to study both connectivity and patch importance in metapopulations (Minor & Urban, 2007, 2008; Urban & Keitt, 2001) and migratory networks (Bauer & Klaassen, 2013; Iwamura et al., 2013; Nicol, Fuller, Iwamura, & Chades, 2015; Taylor & Norris, 2010; Wiederholt et al., 2013), but are not generally suitable to populations that exist on a continuous landscape.

In this study, we first describe the general elements of the network and show how these elements can be represented mathematically. Using both theoretical and real‐world examples, we then demonstrate how, with straightforward modifications to the basic model structure, most, if not all, spatiotemporal population scenarios involving spatially structured populations can be represented using this approach. This includes metapopulations as well as various forms of migration including nomadism, partial, stepping‐stone, and complete migration. Our framework is also flexible enough to include carryover effects, and density dependence, and can accommodate various types of life histories, network sizes, and carrying capacities. It can also be used to investigate interspecific interactions and environmental perturbations. Rather than replacing any existing theory, our work shows how common elements recur across species with disparate movement strategies and how they can be combined under a unified mathematical framework. We are not proposing that our approach can handle spatiotemporal features that current models cannot. Our intent is to present a common language and modeling structure so that it is straightforward to model spatial, temporal, and movement processes in any type of population. A common framework makes it easier to compare different types of populations and study interactions between populations.

## 2 MODEL DEVELOPMENT

### 2.1 Terminology

Network models consist of a set of nodes connected by edges. In the context of populations, *nodes* represent habitats that can have unique “local” attributes, such as habitat size, habitat quality, and density dependence. These attributes not only affect dynamics within that node but potentially other nodes in the network through the movement of individuals between nodes. In addition to unique attributes within a node, nodes can also be classified into *sets* that share attributes. For example, there may be a set of breeding nodes in which individuals can reproduce and a set of nonbreeding nodes in which individuals only survive or die.

*Edges* connect nodes and represent the potential for movement at each time step. They are the elements in the model that define the spatial structure of the system. In addition to connecting nodes, edges can be self‐loops where individuals remain in a node from one time step to another. Edges can be *weighted*, which means they are associated with specific attributes. For example, there could be a cost (decreased survival) to move along an edge that is associated with a specific attribute of the edge (e.g., length or distance). Edges can also be *directed*, indicating a direction of movement between nodes, or *undirected*, meaning that movement can occur in both directions.

### 2.2 Model description

In our model, the spatial structure of a population is represented by a weighted and directed network, consisting of *n* nodes. Each node has a set of attributes which vary through time; for node *i* at time *t*, these attributes are denoted by the vector **α**_*i,t*_ (node characteristics can include demographic processes such as survival and reproduction, as well as representing class and age transitions). Similarly, edges have a set of attributes; for the edge between nodes *i* and *j*, the attributes at time *t* are denoted by the vector **β**_*ij,t*_.

To describe the dynamics of our network‐based population, we develop a model with discrete time steps. In a single time step, we update a node's population size based on demographic information within the node, and simulate movement along the edges, which could represent migration, dispersal, or residency (i.e., self‐loops). Thus, within one time period, we include both within‐node dynamics (i.e., survival and reproduction) and between‐node dynamics (i.e., movement and migration).

At time *t*, we denote the population size of node *j* after movement as *N*_*j,t*_. Thus, for all *j* ∈ {1,…,*n*}, the population size of a node at time *t *+ 1 is described by the sum of all individuals that moved to node *j*, or remained at node *j*, after demographic processes have taken place between times *t* and *t *+* 1*:

(1) $$N_{j,t+1}\;=\;\sum_{i=1}^{n}s_{ij,t}\cdot p_{ij,t}\cdot f_{i,t}$$

where *f*_*i,t*_ is the function for updating population size at node *i* given by

(2) $$f_{i,t}\;\equiv \;f\left(N_{i,t},\alpha_{i,t}\right),$$

*p*_*ij,t*_ is the *transition probability* function that specifies the proportion of individuals moving along an edge,

(3) $$p_{ij,t}\;\equiv \;p\left(N_{i,t},\alpha_{i,t},\beta_{ij,t}\right),$$

and *s*_*ij*,*t*_ is the *edge survival probability* function,

(4) $$s_{ij,t}\;\equiv \;s\left(N_{i,t},\alpha_{i,t},\beta_{ij,t}\right).$$

The product in Equation (1), which we denote as *M*_*ij,t*_,

(5) $$M_{ij,t}\;=\;s_{ij,t}\cdot p_{ij,t}\cdot f_{i,t},$$

gives the total number of individuals traveling along the edge from node *i* to *j* at time *t*. Model variables and functions are summarized in Table 1. Note that the model equations given in Equation (1) can be written in matrix form (see Appendix S1).

**Table 1:** Model variables and functions

*N*_*i,t*_	Population size of node *i* at time *t* after movement to (or residency in) the node
**α**_*i,t*_	Vector of node *i*'s characteristics at time *t,* such as carrying capacity, intrinsic growth rate, and habitat quality. Characteristics may depend on time (e.g., breeding season, nonbreeding season)
**β**_*ij,t*_	Vector of characteristics for the directed edge that connects node *i* to node *j* at time *t*. Characteristics may include number of stopover sites, which may depend on time (e.g., fall migration, spring migration)
*f*_*i,t*_ ≡ *f*(*N*_*i,t*_, **α**_*i,t*_)	Function that represents the population size of node *i* at time *t* before movement to other nodes or residency in the same node. The function accounts for node population dynamics such as survival and reproduction. It depends on population size of node *i* and node characteristics
*p*_*ij,t*_ ≡ *p*(*N*_*i,t*_, **α**_*i,t*_, **β**_*ij,t*_)	Function to determine the proportion of node *i*'s occupants that take movement pathway *ij*; depends on population size and characteristics of the starting node, as well as characteristics of the edge. For example, it may be a function of the starting node's population before movement, *f*_*i,t*_
*s*_*ij,t*_ ≡ *s*(*N*_*i,t*_, **α**_*i,t*_, **β**_*ij,t*_)	Function for the probability that individuals survive movement pathway *ij* depends on population size and characteristics of the starting node and edge characteristics. For example, it may be a function of the number of individuals moving along the edge, *p*_*ij,t*_ ∙ *f*_*i,t*_
*M*_*ij,t*_ = *s*_*ij,t*_ *p*_*ij,t*_ *f*_*i,t*_	The total number of individuals traveling along the edge from node *i* to *j* at time *t*

The function *f*_*i,t*_ of Equation (2) updates the population at node *i* and describes the population size of node *i* at time *t* before movement. It depends on the total number of individuals that arrived at the node at time *t, N*_*i,t*_, and node characteristics represented by the vector **α**_*i,t*_. Note that if one is interested in the population size of node *j* after demographic updates but before movement (instead of after movement, as in Equation (1)), then keeping track of *f*_*j,t*_ over time (instead of *N*_*j,t*_) will provide this information.

The proportional movement function, *p*_*ij,t*_, of Equation (3) gives the proportion of the node's occupants that will move along the edge from node *i* to *j* at time *t*. In terms of the network, *p*_*ij,t*_ represents the *weight* associated with the edge connecting node *i* to node *j*. If *p*_*ij,t*_ is zero, then there is no edge connecting nodes *i* and *j* at time *t*. The function *p*_*ij,t*_ depends on the population at the starting node, *N*_*i,t*_, node characteristics, **α**_*i,t*_, and edge characteristics, **β**_*ij,t*_. Density dependence will most likely be expressed in terms of the starting node's population size after demographic updates, that is *f*_*i,t*_. More complex dependencies may also be included, such as delayed density dependence or carry over effects. We require that for all *i* ∈ {1,…,*n*}, the proportion of node *i*'s individuals that use each outgoing edge at a given time step sums to either 0 or 1. The sum is 1 if node *i* has at least one outgoing edge, which could be a self‐loop, at a given time step. The sum is 0 if a node has no outgoing edges; this implies that the node is temporarily unoccupied at that time (e.g., breeding habitats during the nonbreeding season in a migratory species). As the transition probability *p*_*ij,t*_ is time dependent, the probability of moving from node *i* to node *j* in a given time step, or season, is not necessarily the same probability of moving from node *i* to node *j* in a different time step, or season.

The function *s*_*ij*,*t*_ of Equation (4) is the probability that individuals moving along the edge from node *i* to node *j* will survive during time step *t*. This survival probability can be a function of the population at the starting node, *N*_*i,t*_, characteristics of the starting node **α**_*i,t*_, as well as edge characteristics, **β**_*ij,t*_, which may include the proportion of individuals moving along edge *ij* at time *t*,* p*_*ij*,*t*_. Edge characteristics may also include processes such harvest rates, as seen in fall migration for waterfowl. As with Equation (3), density‐dependent survival will most likely be expressed in terms of the node's population size after demographic updates, *f*_*i,t*_.

If the population being modeled has multiple classes, then Equation (1) can be used for different age classes or stages. For instance, suppose both adults (*A*) and juveniles (*J*) are tracked. At each time step and for each node *i*, Equation (1) is solved for both $N_{i,j}^{A}$ and $N_{i,j}^{J}$. In this case, the functions *s*_*ij*,*t*_ *, p*_*ij*,*t*_ and *f*_*i,t*_ may depend on both $N_{i,j}^{A}$ and $N_{i,j}^{J}$ and class‐specific node and edge characteristics. For example, *Ranunculus nodiflorus*,* Anas acuta* (northern pintail), and *Cervus canadensis* (elk) are populations that are modeled with multiple classes (see Results section).

Each time step can represent any length of time, for example, one season or 1 year. Furthermore, the length of the time step can vary within a given model; time steps do not need to be equal in length. The length of the time step is determined by the life history and major stages of the annual cycle of a species. For example, for a typical North American migratory bird that reproduces after spring migration, the first time step could represent the period from the start of the breeding season (immediately after spring migration) to the end of the fall migration and the second time step could represent the period from the start of the winter season (after fall migration) to the end of spring migration. Parameters need adjustment to reflect the underlying biological meaning for any modification in time step structure (e.g., survival should be adjusted to represent the survival over the entire duration of the time step).

### 2.3 Model Features

#### 2.3.1 Features within a node

Each function in the model can be specified to encompass a variety of ecological phenomena relevant to population dynamics. For many populations, density dependence is important for modeling node‐level population dynamics. Here, *f*_*i,t*_, can be specified so that survival or the population growth rate vary with the number of individuals entering that node or by specifying a node‐specific carrying capacity. Many of the typical functions used to represent different patterns of density dependence are straightforward to incorporate here. For the Ricker equation, for example, node characteristics would include the exponential growth rate and carrying capacity: **α**_*i,t*_ = (*r*_*i,t*_, *K*_*i,t*_).

The model also has the flexibility to specify carryover effects, which are events or processes that occur in one time period but have nonlethal effects on individuals in the following time period (Harrison, Blount, Inger, Norris, & Bearhop, 2011; O'Connor, Norris, Crossin, & Cooke, 2014). For example, survival or reproduction in the node could be affected by the amount of energy reserves individuals have at the beginning of the breeding season, which can be specified as a function of conditions in the node occupied during the previous time step. To incorporate carryover effects in the model, a function could be specified in which survival or reproduction in the node decreases with increasing distance traveled during the most recent migration or with the strength of density dependence in the previously occupied node (Betini, Griswold, & Norris, 2013; Norris & Taylor, 2006).

#### 2.3.2 Features in the proportional movement function

The proportional movement function *p* determines what proportion of node occupants utilizes each possible edge. For migratory animals, a simple assumption for this function is heritability of the migratory route (Taylor & Norris, 2010). That is, the same proportion of individuals arriving via a pathway in time *t *− 1 is directed along the same pathway (but in opposite direction) in time *t*, which can be specified as follows:

(6) $$p_{ij,t}\;=\;\frac{M_{ji,t-1}}{N_{i,t}}.$$

Note that this proportion function depends on the number of individuals that moved along edge *ji* in the previous time step, *M*_*ji*,*t*−1_ given in Equation (5), as well as the total number of individuals that arrived at node *i* at time *t, N*_*i,t*_.

The model also has the capability to incorporate adaptive path switching, capturing the ability of individuals to choose movement paths based on the potential fitness payoff. For example, *p*_*ij,t*_ could vary based on the relative per capita growth rate of nodes in the previous time step (*f*_*j,t*−1_/*N*_*j,t*−1_) such that *p*_*ij,t*_ is lower for pathways to nodes with lower relative per capita growth rates. The proportion *p*_*ij,t*_ could also vary according to a function combining the cost of migration (e.g., inverse of distance) to a specific node and the fitness benefits of that node (e.g., fecundity × node‐specific survival).

#### 2.3.3 Features of migration and dispersal survival

The model can be readily modified to include energetic costs of migration or dispersal. For example, *s*_*ij,t*_ could be a function of the length of a movement pathway, the numbers of individuals using the edge, or the number of stopover sites. Such characteristics are described by **β**_*ij,t*_. These migration and dispersal processes could also include density dependence (Morris, 1987, 1989) or carryover effects, such that survival is dependent on the density or habitat quality of node *i* prior to departure from the node (Donaldson et al., 2010).

#### 2.3.4 Specifying the model for particular movement strategies

Our modeling framework can be adapted to a variety of mobile organisms. The distinguishing feature of each population type is the *structure* of its network, and how it changes with each time step. Recall that the movement proportion function, *p*_*ij,t*_, represents the weight of the edge (relative flow of individuals) connecting node *i* to node *j* at time *t*. Thus, the spatial structure of a population can be described by the movement proportion function, *p*. In particular, if edge *ij* is not used during a time step *t*, then *p*_*ij,t*_ is set to zero.

**Figure 1:**
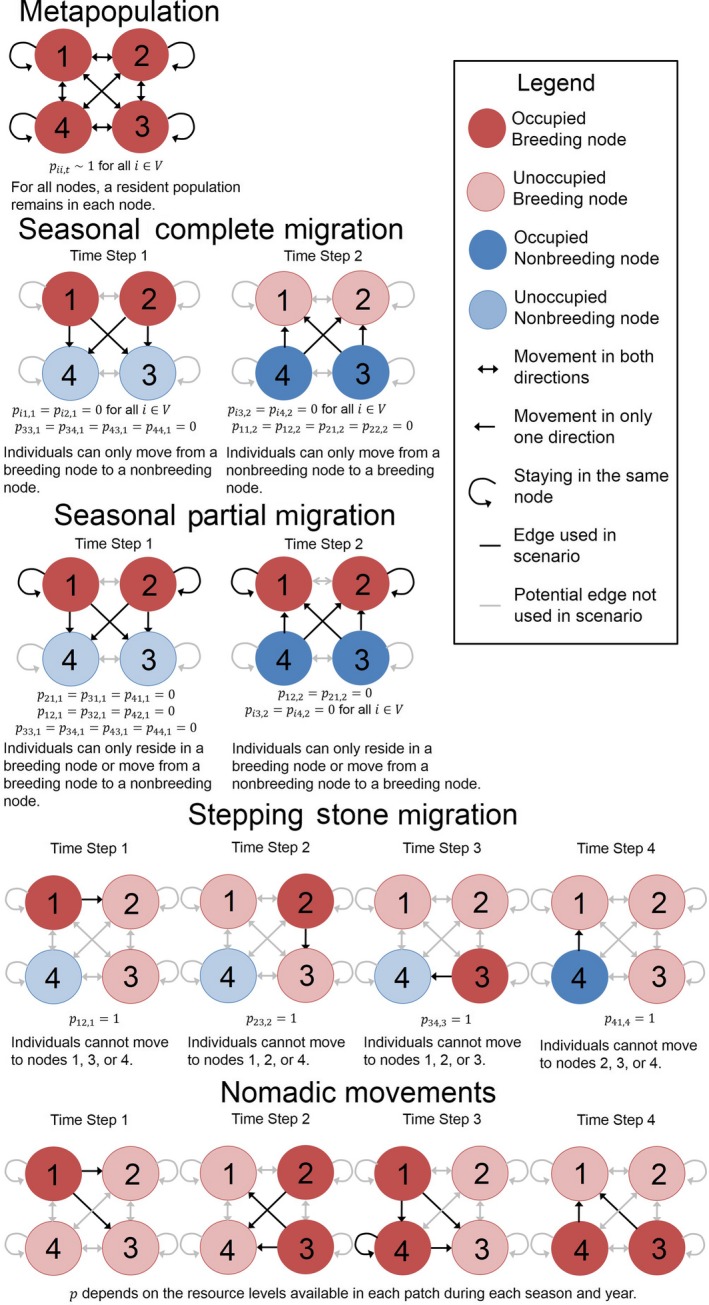
Our flexible framework can be applied to a variety of populations. Illustrated are five examples that exhibit different types of movement patterns: metapopulations, seasonal complete migratory populations, seasonal partial migratory populations, “stepping‐stone” migratory populations, and nomadic populations. These movement patterns are shown using simple four‐node networks with breeding and nonbreeding sites. The number of stationary/migration steps vary with each population, and conditions on the transition probabilities, *p*_*ij,t*_, are described for each time step

We applied our framework to five commonly recognized types of spatially structured populations: metapopulation, seasonal complete migration, seasonal partial migration, “stepping‐stone” migration, and nomadism (Figure 1). We recognize that these strategies are not mutually exclusive; individuals of many species may exhibit multiple strategies depending on the temporal and spatial scale of investigation (Chapman et al., 2014; Jonzén, Knudsen, Holt, & Sæther, 2011), and our modeling framework is flexible enough to allow for a single species to be modeled using multiple alternative network specifications.

In specifying functions *f*,* p*, and *s* for a movement strategy, it is useful to classify a node as a breeding or nonbreeding node. For this discussion, we represent the set of all nodes as *V*. We denote *V*_*B*_ ⊆ *V* as the set of breeding nodes and *V*_*NB*_ = *V *− *V*_*B*_ as the set of nonbreeding nodes. Therefore, node *i* ∈ *V*_*B*_, refers to a breeding node, *i* ∈ *V*_*NB*_ refers to a nonbreeding node, and *i* ∈ *V* refers to a node of any type in the network.

### 2.4 Spatially structured population types

#### 2.4.1 Metapopulation

In its most basic form, the modeling framework can represent a metapopulation, where *f*_*i,t*_ represents reproduction and survival in a node. During a time step, the portion of the population that remains in each node is represented by a self‐loop in the network structure. Thus, if some individuals in node *i* remain there for the next time step, then *p*_*ii,t*_ is nonzero and *s*_*ii,t*_ is the resident survival rate. In the same time step *t*, a portion of individuals may disperse to other nodes, such that *p*_*ij,t*_ is nonzero for *i ≠ j*. Survival during dispersal is *s*_*ij,t*_. For a typical metapopulation, all nodes are breeding nodes (*V* = *V*_*B*_), and dispersal to other nodes is infrequent. That is, the proportion of the population that are residents is usually close to one (*p*_*ii,t*_ ~ 1 for all *i*), and the proportion of the population that disperses to other nodes is close to zero (*p*_*ij,t*_ ≪ 1 for *i *≠ *j*) for any given time step.

There are numerous examples of species that typically occur in metapopulations, including *Drepanotrema depressissimum* (tropical freshwater snails; Lamy et al., 2013)*, Tetrax tetrax* (little bustards; Bretagnolle & Inchausti, 2005)*,* and *Lithobates sylvaticus* (wood frogs; Peterman, Rittenhouse, Earl, & Semlitsch, 2013), although species recognized as having a “classic metapopulation” structure are rare (Fronhofer, Kubisch, Hilker, Hovestadt, & Poethke, 2012). Metapopulations can be modeled many ways using our framework, such as making nodes ephemeral by disallowing any survival or reproduction in a node during certain time periods, as in ponds used by tropical freshwater snails (Lamy et al., 2013). Source‐sink dynamics can be modeled by altering node‐specific survival and reproduction to create sources and sinks. Finally, density‐dependent habitat selection (Morris, 1987, 1989) could be modeled by making *p*_*ij,t*_ a function of node carrying capacity and population size.

#### 2.4.2 Seasonal complete migration

For a seasonal, complete migratory pattern, the network is bipartite and consists of two disjoint sets of breeding nodes, *V*_*B*_, and nonbreeding nodes, *V*_*NB*_. No direct movement occurs between breeding nodes nor between nonbreeding nodes. That is, individuals only move from a breeding node to a nonbreeding node or from a nonbreeding node to a breeding node. Neotropical migrants such as *Hylocichla mustelina* (wood thrush; Stanley et al., 2015) and *Setophaga ruticilla* (American redstarts; Norris et al., 2006) exhibit this type of migration, moving from breeding sites in the United States and Canada to overwintering grounds in Central America and the Caribbean.

In the example of a seasonal complete migration network depicted in Figure 1, the first time step begins during the breeding season and ends with completion of migration to the nonbreeding nodes (*p*_*ij,1*_ is nonzero only if *i* ∈ *V*_*B*_ and *j* ∈ *V*_*NB*_). The second time step begins during the nonbreeding season and ends with subsequent migration back to the breeding nodes (*p*_*ij,2*_ is nonzero only if *i* ∈ *V*_*NB*_ and *j* ∈ *V*_*B*_). There are no year‐round residents and no movement between breeding habitats nor between nonbreeding habitats.

#### 2.4.3 Partial migration

For a seasonal, partial migratory pattern, year‐round residents and migratory individuals occur in one or more nodes. Species such as *Tadarida brasiliensis mexicana* (Mexican free‐tailed bats) display this type of migration, where the majority of males and some females remain on the nonbreeding grounds year‐round (Federico et al., 2008; McCracken & Gassel, 1997). As another example, some subpopulations of *Cervus canadensis* (elk) stay in the breeding grounds at all times and forgo migration to overwintering grounds (Middleton et al., 2013).

In our example of a partial migration network (Figure 1), the first time step begins with the entire population in breeding nodes during the breeding season. The time step ends after a portion of the population migrates to the nonbreeding nodes (i.e., *p*_*ij,*1_ is nonzero for some *i* ∈ *V*_*B*_ and *j* ∈ *V*_*NB*_ *)*. The second time step begins in the nonbreeding season and ends with the migratory individuals moving back to the breeding nodes (i.e., *p*_*ij,*2_ is nonzero for some *i* ∈ *V*_*NB*_ and *j* ∈ *V*_*B*_ *)*. Partial migration is modeled by a self‐loop (i.e., *p*_*ii,t*_ > 0 for some *i* ∈ *V*) for those breeding areas and nonbreeding areas where some individuals remain as year‐round residents.

#### 2.4.4 Stepping‐stone migration

Our framework can also be applied to more complex migratory patterns. In this example, we illustrate a “stepping‐stone” migration system. In this pattern, individuals travel through a series of nodes, one by one throughout their annual cycle. Many migratory bird species follow a stepping‐stone pattern, as individuals stop to refuel at staging areas between breeding and nonbreeding grounds (Buler & Dawson, 2014). Some insect species also display this migratory pattern with *Danaus plexippus* (monarch butterfly) as a well‐known example (Chapman et al., 2014; Prysby & Oberhauser, 2004).

For the example stepping‐stone network illustrated in Figure 1, there is directed movement between successive breeding nodes followed by movement to a nonbreeding node within one annual cycle. Only one edge is used per time step. That is, migration only occurs from one habitat to one other habitat. Consequently, edge transition probabilities are either zero or one. The first time step begins with all individuals occurring in node 1 for their first breeding season of the year and ends upon completion of migration to node 2 (*p*_*12,*1_ = 1). The second time step begins with all surviving individuals initiating their second breeding season in node 2 and ends upon completion of migration to breeding node 3 (*p*_*23,2*_ = 1). All surviving individuals breed for the third time step in node 3 and then migrate to nonbreeding node 4 (*p*_*34,*3_ = 1). The last time step begins with all surviving individuals in nonbreeding node 4 and ends with completion of migration to breeding node 1 (*p*_*41,*4_ = 1).

#### 2.4.5 Nomadism

For nomadism, movement to any site during any time step is permitted. Nomadism differs from migration in that although the movements do correspond with environmental fluctuations, interannual variability is inconsistent and thus the timing of nomadic movements varies from year to year. Whereas metapopulations are characterized by rare movements (i.e., *p*_*ij,t*_ **~ 0 for *i *≠ *j*), nomadic individuals are more likely to move between nodes multiple times per year. Nomadic species are animals that rely on food sources that are extremely ephemeral, exemplified by desert dwellers (e.g., *Polytelis alexandrae* (princess parrot); Jonzén et al., 2011; Cottee‐Jones, Matthews, & Whittaker, 2016).

In the example nomadic network (Figure 1), all nodes are breeding nodes (*V* = *V*_*B*_). Unlike seasonal and stepping‐stone migration, none of the time‐specific edge transition probabilities *p*_*ij,t*_ are set to 0 or 1 unless so determined by the resource levels available in each node during each season and year. This can be modeled stochastically or by letting *p*_*ij,t*_ be a function of the population size at node *i* as well as characteristics and population sizes of other nodes.

## 3 RESULTS

**Figure 2:**
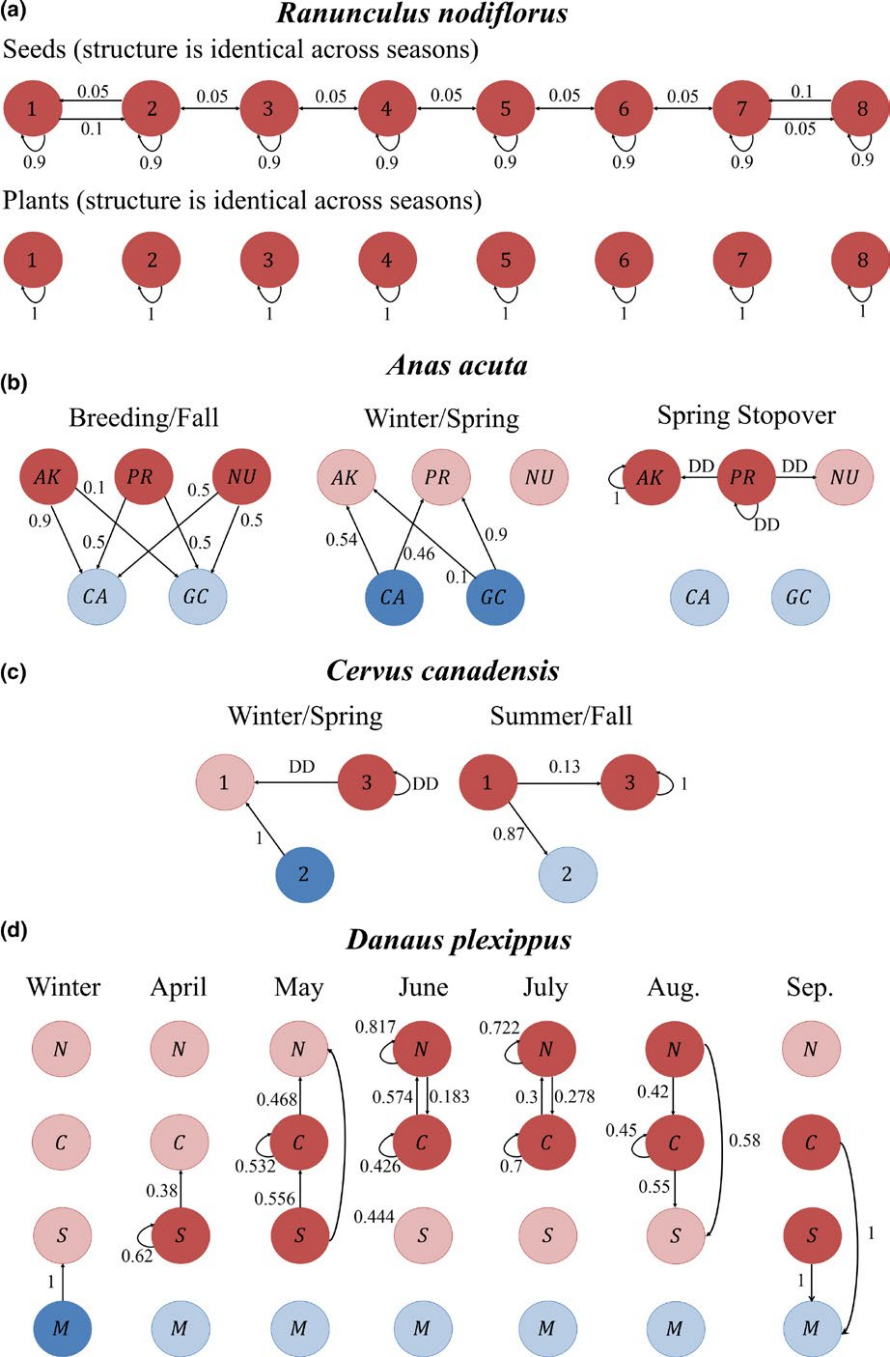
Our modeling framework is applied to four example species showing a wide range of life histories, movement patterns, and carrying capacities. The network structure and edge transition probabilities for each population are shown, where DD indicates a density‐dependent transition probability. (a) *Ranunculus nodiflorus* is modeled as a metapopulation with eight nodes, three seasons, and two age classes (seeds and plants). Seed and plant transition probabilities differ. As plants do not disperse, the network is disconnected and all plants remain in their node. (b) *Anas acuta* (northern pintail) exhibits seasonal complete migration. The population is modeled with three breeding nodes and two nonbreeding nodes in three seasons. There and two classes, females and males, with two age classes for each sex, juveniles and adult. Edge transition probabilities are the same for all classes. (c) *Cervus canadensis* (elk) comprises a partial seasonal migratory population with three nodes and two seasons. The female population is modeled with two age classes, adults and juveniles. The two classes have the same constant transition probabilities, but different density‐dependent transition probabilities. (d) *Danaus plexippus* (monarch butterfly) has a stepping‐stone movement pattern. The population is modeled using one class, four nodes and seven seasons. See Appendix S2 and Sample et al. (2017) for model details, outcomes, parameterization, and computer code for each species

We illustrated above how our modeling framework can be adapted to a wide variety of mobile organisms using theoretical examples. We now demonstrate how our framework can be formally implemented by applying it to four example species showing a wide range of life histories, movement patterns, and carrying capacities. Our examples include a plant (*Ranunculus nodiflorus*) modeled as a metapopulation, a bird (*Anas acuta*) exhibiting seasonal complete migration, a mammal (*Cervus canadensis*) comprising a partial seasonal migratory population, and an insect (*Danaus plexippus*) that has as a stepping‐stone movement pattern. Table 2 gives a summary of the four models. Figure 2 illustrates their network structure. Details are provided here on how the four models were parameterized. A full description of each model, as well as additional model results, can be found in Appendix S2. General R code (version 3.2.1) was created for the general network framework of Equation (1) and then adapted for each of the four examples. The code as well as files with model parameters can be found in Sample et al. (2017).

**Table 2:** Model summary for species‐specific example populations

Attribute	*Ranunculus nodiflorus*	*Anas acuta*	*Cervus canadensis*	*Danaus plexippus*
Movement system	Metapopulation	Seasonal complete migration	Seasonal partial migration	Stepping‐stone migration
Number of nodes	8	3 breeding, 2 nonbreeding	3	3 breeding, 1 nonbreeding
Number of time steps in cycle	3	3	2	7
Recruitment	Locally density‐dependent	Locally density dependent	Locally density dependent	Locally density dependent
Survival	Constant	Node and edge specific, locally density dependent	Node‐specific, locally density dependent	Node and edge specific
Movement probabilities	Constant	Edge specific, logistic density‐dependent function for some in spring	Edge specific	Edge specific
Special features	Age structure	Sex specific, age structure, harvest	Age structure, female only	Multiple generations within annual cycle
Carrying capacity	800	5,500,000	3600	Unknown
Key reference	Noël et al. (2013)	Mattsson et al. (2012)	Middleton et al. (2013)	Flockhart et al. (2015)

### 3.1 *Ranunculus nodiflorus* (metapopulation)

To illustrate the application of our framework to a metapopulation, we modeled *Ranunculus nodiflorus* (buttercup family), which is a rare and endangered annual plant that occurs in Spain, Portugal, and France (Noël, Machon, & Robert, 2013). The plant grows only in ponds, reproduces by selfing (Kircher, Ferdy, Andalo, Colas, & Moret, 2003), produces seeds during April and May, and then dies soon after reproduction (Noël et al., 2013). Seeds float and disperse along water corridors that arise during flooding connecting adjacent ponds, and thus, they operate as a typical metapopulation (Kircher et al., 2003; Noël et al., 2013). Some seeds germinate in autumn and others germinate in spring. We model the system as an age‐structured metapopulation with eight nodes based on a representative chain of ponds (Figure 2a). The two age classes are as follows: seeds, denoted by a superscript *S*, and plants, denoted by a superscript *P*. We track both classes through three times steps, or seasons, within an annual cycle: summer, autumn/winter, and spring.

The nodal update function (Equation (2)) for seeds, $f_{i,t}^{S}\;\equiv \;f\left(N_{i,t}^{S},N_{i,t}^{P},\alpha_{i,t}\right)$ is given by

fi,tS=si,tS·Ni,tS⏟seedsthatsurvive+Ri,t·Ni,tP⏟newseedsfromplants−Ti,t·si,tS·Ni,tS⏟seedsthattransititiontoplants

Seed survival rates, $s_{i,t}^{S}\;=\;0.7$, are constant across seasons and identical across nodes, *R*_*i*,*t*_ is the reproductive rate of plants producing seeds and equal to 11.5 in the summer and zero otherwise, and *T*_*i*,*t*_ represents the germination rate or transition rate of seeds to plants, which is zero in the summer, 0.569 in the autumn/winter, and 0.995 in the spring. The nodal update function for plants, $f_{i,t}^{P}\;\equiv \;f\left(N_{i,t}^{S},N_{i,t}^{P},\alpha_{i,t}\right)$, is given by

fi,tP=si,tP·Ni,tP⏟plantsthatsurvive+Ψi,t·Ti,t·si,tS·Ni,tS⏟seedsthatsuccessfullytransititiontoplants

We assume that, at each node, no plants survive the summer after they produce seeds ($s_{i,t}^{P}\;=\;0$) and that all plants survive in the autumn/winter and spring seasons ($s_{i,t}^{P}\;=\;1$). The second term is the product of seeds that survive, $s_{i,t}^{S}N_{i,t}^{S}$, the seed germination rate, $T_{i,t}$, and the density‐dependent postgermination survival rate $\Psi_{i,t}$ is given by

$$\Psi_{i,t}\;=\;\psi_{i,t}\exp\left(-\frac{N_{i,t}^{P}\;+\;T_{i,t}\cdot s_{i,t}^{S}\cdot N_{i,t}^{S}}{K_{i}}\right)$$

*K*_*i*_ = 100 is the carrying capacity at node *i* and the maximum germination rate, $\psi_{i,t}$, is 0.34 in the autumn/winter and 1 in the spring. Thus, the vector of node characteristics is $\alpha_{i,t}\;=\;\left(s_{i,t}^{S},s_{i,t}^{P},T_{i,t},R_{i,t},K_{i},\psi_{i,t}\right)$ representing all node‐specific parameters. We assume edge transition probabilities (Equation (3)) are not season dependent and that seeds remain at the node 90% of the time and disperse to any adjacent node with equal probability. Plants do not move between nodes so that *p*_*ij,t*_ = 1 if *i *= *j* and 0 otherwise (Figure 2a). We further assume an edge survival probability of 1 for every edge and at every time step (Equation (4)).

**Figure 3:**
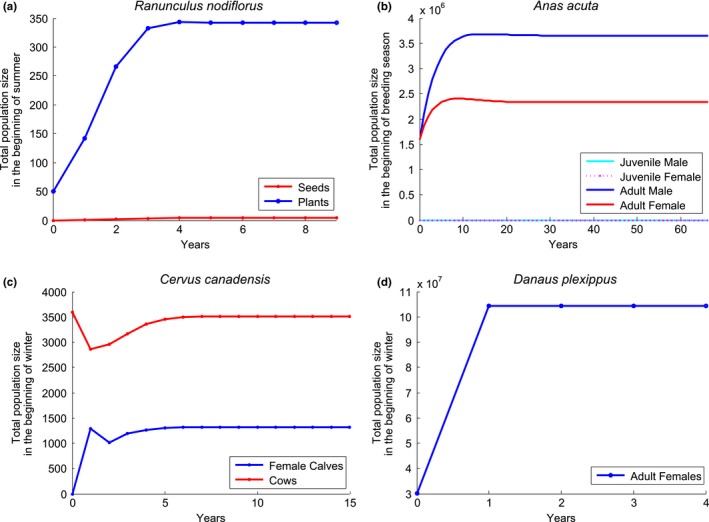
We demonstrate that our model can be applied to a variety of populations. We show simulated population dynamics for all four species example species by running the code provided in Sample et al. (2017). (a) After 9 years, the small initial population of plants (*Ranunculus nodiflorus*) disperses to all ponds in the network and sustain a persistent population. (b) The pintail model converged to a steady‐state solution after 66 years, with a breeding population of 5.98 million, which is comparable to the results found by Mattsson et al. (2012) in the absence of harvest. Note that there are more males than female and no juveniles in the beginning of the breeding season. (c) After 16 years, the elk model reached a steady state. (d) The monarch model converged to a steady state after 4 years

In the model, ponds containing the plant in an initial 1999 study (Kircher et al., 2003) were assigned a small initial population of 10 in the beginning of the summer. Initial seed population was set to 0. After 9 years, the small initial population of plants disperse to all ponds in the network and sustain a small, but persistent population (Figure 3a). Table 3 shows equilibrium population values for each season and each class. Table 4 presents the equilibrium population distribution in each of the ponds, which depends on the season.

**Table 3:** Equilibrium population of plants and seeds at the beginning of each season for *Ranunculus nodiflorus*

Season	Seeds	Plants
Summer	4	342
Autumn/Winter	3,942	0
Spring	1,189	75

**Table 4:** Equilibrium population distribution of plants at the beginning of each season for *Ranunculus nodiflorus*

Node	Summer	Autumn/Winter	Spring
Ponds 1 and 8	0.126	0	0.130
Ponds 2 and 7	0.124	0	0.120
Ponds 3–6	0.125	0	0.125

### 3.2 *Anas acuta* (complete migration)

The northern pintail is an example of a population that performs seasonal complete migration. The northern pintail is widely distributed in wetland regions; it breeds in the northern areas of North America, Europe, and Asia and winters close to the equator. Some individuals use a stopover site during spring migration. We illustrate how the sex‐specific and age‐structured model for the North American population presented in Mattsson et al. (2012) can be translated to our network framework. Reproduction, winter survival, and one of the movement probabilities are density dependent, and the model accounts for birds killed by hunters each fall, making one of the edge survival probabilities density dependent (Figure 2b).

The model consists of a network of five nodes with a set of three breeding nodes, Alaska (*AK*), Prairie Pothole (*PR*), and Northern Unsurveyed (NU)*,* enumerated 1–3, and a set of two wintering nodes, California (*CA*) and Gulf Coast (*GC*), enumerated 4 and 5. The *PR* node also serves as a stopover site during spring migration. Thus, we divide the annual cycle into three time steps: breeding/fall, winter/spring, and spring stopover. Time steps vary in length, depending on the season. The population is modeled using two classes, females (*F*) and males (*M*), with two age classes for each sex, juveniles (*J*) and adults (*A*). The main reason to group the individuals in four different categories is differential node and edge survival rates. Let $N_{i,t}^{A∙}$ be the number of adult males or females and $N_{i,t}^{J∙}$ be the number of juvenile males or females in node *i* at time *t*, where the superscript indicates the sex class: ∙∈{M,F}. We define the vector $N_{i,t}\;=\;\left(N_{i,t}^{\mathrm{AF}},N_{i,t}^{\mathrm{AM}},N_{i,t}^{\mathrm{JF}},N_{i,t}^{\mathrm{JM}}\right)$, whose elements consist of the population sizes for the four classes at node *i* and time *t*.

The function (Equation (2)) that represents adult survival and transitions into the adult stage for each node *i* at time step *t*, $f_{i,t}^{A∙}\;\equiv \;f\left(N_{i,t},\alpha_{i,t}\right)$, is given by

fi,tA∙=si,tA∙·Ni,tA∙⏟adultsthatsurvive+Ti,t·si,tA∙·Ni,tJ∙·⏟juvenilesthattransitiontoadultsandsurvive

*T*_*i*,*t*_ represents the transition from juveniles to adults, which is 1 in the winter/spring season and 0 otherwise. Adult node‐specific survival, $s_{i,t}^{A∙}$, is constant during the breeding/fall season (0.81 for females and 0.98 for males) and stopover step (1 for both sexes). In the winter (i.e., posthunting), it depends on density and other node characteristics (eqn 3 of Mattsson et al. (2012)). The nodal update function for juveniles, $f_{i,t}^{J∙}\;\equiv \;f\left(N_{i,t},\alpha_{i,t}\right)$, exclusively represents the addition of offspring in node *i* at time *t*,

fi,tJ∙=Ri,t·si,tAF·Ni,tAF⏟juvenilesborntoadultfemales

where *R*_*i*,*t*_ is the reproduction rate, which is nonzero during the breeding/fall season and depends on total nodal population size and other node characteristics (eqn 2 of Mattsson et al. (2012)). Edge transition probabilities (Equation (3)) do not depend on the sex or age class, $p_{ij,t}^{A∙}\;=\;p_{ij,t}^{J∙}\;\equiv \;p\left(N_{i,t},\alpha_{i,t},\beta_{ij,t}\right)$ and are constant except during the spring stopover step when the probability is density dependent when the origin node is *PR* (Figure 2b). In this case,

$$p_{2j,t}^{A∙}\;=\;p_{2j,t}^{J∙}\;=\;\left\{\begin{array}{cc} 1-\frac{\psi_{2}^{\max}}{1\;+\;e^{-Y_{t}}}, & j\;=\;2\\ \frac{\psi_{2j}\cdot \psi_{2}^{\max}}{1\;+\;e^{-Y_{t}}}, & j\;\neq \;2 \end{array}\right.$$

where

$$Y_{t}\;=\;\delta_{20}\;+\;\delta_{21}\left(N_{2,t}^{F}\;+\;N_{2,t}^{M}\right)\;+\;\delta_{22}P_{2}.$$

Edge survival probabilities (Equation (4)), $s_{ij,t}^{A∙}\;\equiv \;s\left(\beta_{ij,t}\right)$ and $s_{ij,t}^{J∙}\;\equiv \;s\left(\beta_{ij,t}\right)$ are constant and only sex dependent during fall migration when hunting takes place. Given the above parameterization, the vector of node characteristics is $\alpha_{i,t}\;=\;\left(s_{i,t}^{F},s_{i,t}^{M},P_{i},a_{\mathrm{ik}},\delta_{\mathrm{ik}},\psi_{i}^{\max},b_{i0},b_{i1},s_{i\min}^{F},s_{i\max}^{F},s_{i\min}^{M},s_{i\max}^{M}\right)$ for k∈{0,1,2} and the vector of edge characteristics is given by $\beta_{ij,t}\;=\;(s_{ij,t}^{A},s_{ij,t}^{J},K_{ij,t}^{\mathrm{AM}},K_{ij,t}^{\mathrm{AF}},K_{ij,t}^{\mathrm{JM}},K_{ij,t}^{\mathrm{JF}},\psi_{\mathrm{ij}})$.

The model has an initial population of $N_{0}^{\mathrm{AF}}\;=\;N_{0}^{\mathrm{AM}}\;=$ [465000,986850,160650,0,0] and zero population for juveniles (Mattsson et al., 2012). The model converged to a steady‐state solution after 66 years, with a breeding population of 5.98 million (Figure 3b), which is comparable to the results found by Mattsson et al. (2012) in the absence of harvest. Table 5 shows the equilibrium population. Equilibrium population distribution (Table 6) demonstrates the relative node importance. The highest proportion of the population is located in AK, CA, and PR during the breeding/fall, winter/spring, and spring stopover seasons, respectively. Pathway importance can be assigned by calculating the proportion of migrants using a path. Table 7 presents the average annual flux, or proportion of migrants, using each path in the pintail model. Here, we see that the path representing migrants who remain in AK between the stopover and breeding season and the path representing the transition from AK to CA have the highest flux.

**Table 5:** Equilibrium population of males, females, and juveniles at the beginning of the season for *Anas acuta*

Season	Adult female	Adult male	Juvenile female	Juvenile male
Breeding/fall	2,332,055	3,651,902	0	0
Winter/spring	1,696,110	3,213,489	985,778	985,778
Spring stopover	2,332,055	3,651,902	0	0

**Table 6:** Equilibrium population distribution at each node at the beginning of each season for *Anas acuta*. The five nodes are Alaska (AK), Prairie Pothole (PR), Northern Unsurveyed (NU), California (CA), and Gulf Coast (GC)

Node	Breeding/Fall	Winter/Spring	Spring Stopover
AK	0.4187	0	0.3875
PR	0.3003	0	0.6125
NU	0.2810	0	0
CA	0	0.6716	0
GC	0	0.3284	0

**Table 7:** Equilibrium pathway flux, averaged across seasons for *Anas acuta*. Here, pathway flux is the proportion of migrants using a pathway and the row indicates the origin node and column is the destination node. The five nodes are Alaska (AK), Prairie Pothole (PR), Northern Unsurveyed (NU), California (CA), and Gulf Coast (GC)

	AK	PR	NU	CA	GC
AK	0.129	0	0	0.128	0.013
PR	0.010	0.100	0.094	0.056	0.056
NU	0	0	0	0.040	0.040
CA	0.118	0.100	0	0	0
GC	0.012	0.104	0	0	0

### 3.3 *Cervus canadensis* (Partial Migration)

Elk are large mammals that occur across North America. The best‐studied populations occur in, and adjacent to, Yellowstone National Park, where the abundance of elk has been monitored for decades (Middleton et al., 2013). Elk near Cody, Wyoming comprise a partial seasonally migratory population where one group of elk remain resident year‐round in areas east of Yellowstone National Park and another group migrates seasonally from a shared overwintering grounds to breeding grounds in Yellowstone National Park (Middleton et al., 2013). Our model accounts for density‐dependent variation in recruitment and survival at the nodes (Figure 2c).

The female elk population is modeled with a network of three nodes and two age classes, juveniles (*J*) and adults (*A*). Juveniles are elk less than 1 year old. Furthermore, we assume the ratio of adult females to adult males is 4:1 (Mack & Singer 1993) and the ratio of female juveniles to male juveniles is 1.5:1 (Houston 1982). We divide the annual cycle into two time steps: winter/spring and summer/fall. Nodes 1–3 are labeled Yellowstone, Nonbreeding migratory, and Cody year‐round.

The function of Equation (2), $f_{i,t}^{A}\;\equiv \;f(N_{i,t}^{A},N_{i,t}^{J},\alpha_{i,t})$ represents adult survival with rate $s_{i,t}^{A}$ and transitions into the adult group with rate *T*_*t*_ for node *i* at time *t* and is given by:

fi,tA=si,tA·Ni,tA⏟adultsthatsurvive+Tt·si,tJ·Ni,tJ·⏟survivingjuvenilesthattransitiontoadults

We assume female juveniles transition to adults every summer; therefore, *T*_*t*_ = 1 during the summer/fall and zero otherwise. A previous model by Taper (2002) proposed a density‐dependent adult survival, so we set the seasonal rate to be

$$s_{i,t}^{A}\;=\;\sqrt{\exp\left(-0.219\cdot {\left(\frac{N_{i,t}^{A}\;+\;N_{i,t}^{J}}{K_{i}^{A}\;+\;K_{i}^{J}}\right)}^{3.77}\right)}.$$

Juvenile survival, $s_{i,t}^{J}$, is constant during the winter/spring season and density dependent in the summer/fall season (Singer et al. 1997):

$$s_{i,t}^{J}\;=\;\left\{\begin{array}{cc} \;\;\;\;\;\;0.72 & t\;=\;0,2,4,\ldots (\text{winter}/\text{spring})\\ 0.65\exp\left(1-\frac{N_{i,t}^{A}\;+\;N_{i,t}^{J}}{K_{i}}\right) & t\;=\;1,3,5,\ldots (\text{summer}/\text{fall}) \end{array}\right.$$

Above, *K*_*i*_ is the carrying capacity of node *i*, which we assume to be proportional to area (details on parameterization can be found in Appendix S2). The function, $f_{i,t}^{J}\;\equiv \;f\left(N_{i,t}^{A},N_{i,t}^{J},\alpha_{i,t}\right)$, which represents juveniles’ survival, recruitment, and transitions out of this group, is given by

fi,tJ=(1−Tt)·si,tJ·Ni,tJ⏟survivingjuvenilesthatdonottransitiontoadults+0.6·ri,t·si,tA·Ni,tA⏟femalecalvesborn

where reproduction rates, *r*_*i*,*t*_, are estimated by the proportion of pregnant elk, using a weighted average for all age classes presented in Figure 3 of Middleton et al. (2013), and assumes that cows do not have twins. Reproduction rate is higher for residents (0.86) than for the migratory (0.68) subpopulation, and it only occurs during the summer/fall time step. The coefficient 0.6 represents the proportion of calves that are female. Given the above parameterization, the vector of node characteristics is given by $\alpha_{i,t}\;=\;\left(s_{t}^{0},r_{\mathrm{it}},K_{i}^{A},K_{i}^{J}\right)$.

Edge transition probabilities, $p_{ij,t}^{∙}\;\equiv \;p\left(N_{i,t}^{∙},\alpha_{i,t},\beta_{ij,t}\right)$, for juveniles and adults are constant during fall migration, but density dependent during spring migration (Figure 2c). This density dependence accounts for the inheritance of a movement pathway: A resident elk will remain a resident and a migratory elk will remain migratory (Equation (6)). The density dependence is modeled using

$$p_{33,t}^{∙}\;=\;\frac{M_{33,t-1}^{∙}}{N_{3,t}^{∙}}$$

$$p_{31,t}^{∙}\;=\;1-\frac{M_{33,t-1}^{∙}}{N_{3,t}^{∙}}.$$

Note that $M_{33,t-1}^{∙}$ is given in Equation (4) and represents the number of individuals (of the specified class) that were residents in node 3 in the previous time step. For the winter/spring season, all individuals in node 2 migrate to node 1, $p_{21,t}^{∙}\;=\;1$ and for the summer/fall season the resident elk population in node 3 will remain there, $p_{33,t}^{∙}\;=\;1$. We assume migration mortality is taken into account at the nodes and therefore set edge survival to one: $s_{ij,t}^{∙}\;=\;1$ for all *t* and all i,j∈{1,…,n}.

The model is simulated with an initial population of $N_{0}^{A}\;=\;\left[0,1427,2173\right]$ at the start of winter and zero calves. The model converged to a steady state after 16 years (Figure 3c). The calf:cow ratio before breeding in the beginning of summer is 0.31 for the migratory subpopulation and 0.37 for the resident subpopulation. The calf:cow ratio at the beginning of winter is 0.36 in node 2 (migratory subpopulation) and 0.40 in node 3 (migratory and residents). These results align with observations from the mid‐1990s (Middleton et al., 2013). Table 8 shows the equilibrium population numbers. The equilibrium population distribution (Table 9) shows that the highest proportion of the population resides in the year‐round node for both seasons, indicating that management actions at this location could have a large impact. Average pathway flux is shown in Table 10. Here, we see that the pathway representing the resident population remaining in the year‐round node has the highest flux, so management actions aimed at the resident population may impact the highest number of animals.

**Table 8:** Equilibrium population of adult and juvenile females for *Cervus canadensis* at the beginning of each season

Season	Juveniles	Adults
Winter/Spring	1,318	3,507
Summer/Fall	949	2,968

**Table 9:** Equilibrium population distribution at each node during the beginning of each season for *Cervus canadensis*

Node	Winter/Spring	Summer/Fall
Yellowstone	0	0.42
Nonbreeding migratory	0.35	0
Cody year‐round	0.65	0.58

**Table 10:** Equilibrium pathway flux averaged across seasons for *Cervus canadensis*. Here, pathway flux is the proportion of migrants using a pathway, where the row indicates the origin node and column is the destination node

Node	Yellowstone	Nonbreeding migratory	Cody year‐round
Yellowstone	0	0.18	0.03
Nonbreeding migratory	0.18	0	0
Cody year‐round	0.03	0	0.59

### 3.4 *Danaus plexippus* (stepping‐stone migration)

Each autumn, monarch butterflies in eastern North America migrate from breeding areas in the northern USA and southern Canada to nonbreeding areas in central Mexico. At the end of the 6‐month nonbreeding season, monarchs begin to mate and migrate north in March to breeding grounds in the southern USA. Remigrating butterflies lay eggs and die, whereupon their eggs develop into caterpillars and then butterflies, which continue to fly north and recolonize the entire breeding distribution in successive breeding generations until September. The last generation of monarchs eclose in a nonreproductive state (diapause) and migrate south en masse to the overwintering colonies in Mexico. The recolonization over multiple breeding generations and return migration to the nonbreeding grounds is represented as a stepping‐stone migration pattern. We convert the model presented in Flockhart, Pichancourt, Norris, and Martin (2015) to our network‐based model, which accounts for density‐dependent recruitment at the nodes (Figure 2d).

The female monarch population is modeled using a network of four nodes representing regions of eastern North America: Mexico (*M*), South (*S*), Central (*C*), and North (*N*), enumerated 1–4, respectively. Mexico is considered a wintering node, and the other three nodes are breeding nodes. An annual cycle consists of seven time steps: Winter, April, May, June, July, August, and September.

The nodal update function of Equation (2), $f_{i,t}\;\equiv \;f\left(N_{i,t},\alpha_{i,t}\right)$ accounts for survival and reproduction:

fi,t=siA·Ni,t⏟adultsthatsurvive+siA·siP·siL·E·Ni,t⏟eggsthatsurviveandtransitiontoadults

and the vector of node characteristics is $\alpha_{i,t}\;=\;\left(s_{i}^{A},s_{i}^{P},m_{i}\right)$. Here, E=268 is the number of eggs per female per month. Adult survival, $s_{i}^{A}$, is 0.939 in the winter and 0.308 otherwise, and pupal survival, $s_{i}^{P}$, is 0.849 at node *i* (Flockhart et al., 2015). Larval survival is dependent on egg density per milkweed stem at node *i* (Flockhart, Martin, & Norris, 2012). Edge transition probabilities of Equation (3), *p*_*ij*,*t*_ vary across seasons but are assumed to be constant, not density dependent, each year (Figure 2d). Transition probabilities are derived from Table S3 in Flockhart et al. (2015). The edge survival probabilities, *s*_*ij*.*t*_, given in Equation (4) are constant for a given time step *t*. Survival probabilities were derived from an expert elicitation exercise as presented in Flockhart et al. (2015). The model has an initial population of $N_{0}\;=\;\left[28250000,0,0\right]$. The model converged to a steady‐state solution after 4 years (Figure 3d). Table 11 shows the equilibrium population numbers at the beginning of each season. Equilibrium population distribution at the nodes (Table 12) shows the relative importance of Mexico and South regions, as 100% of the population resides in each node during the Winter and April season, respectively. During the later months of August and September, the population is more evenly split among the occupied nodes.

**Table 11:** Equilibrium population numbers at the beginning of each season for adult *Danaus plexippus*

Season	Adult monarchs
Winter	104,369,878
April	50,678,510
May	65,711,517
June	86,725,288
July	134,481,626
August	142,239,303
September	128,489,061

**Table 12:** Equilibrium population distribution at each node at the beginning of each season for *Danaus plexippus*

Node	Winter	April	May	June	July	August	September
Mexico	1.000	0	0	0	0	0	0
South	0	1.000	0.690	0	0	0	0.484
Central	0	0	0.310	0.617	0.342	0.508	0.516
North	0	0	0	0.383	0.658	0.492	0

## 4 DISCUSSION

We provided a network‐based framework that can be applied to metapopulations, a wide range of migratory patterns, and other spatially structured populations. This is, to our knowledge, the only modeling framework that is flexible enough to accommodate different types of spatially structured populations (Esler, 2000; Taylor & Hall, 2012). It can be adapted to accommodate different forms of class or age structures, various forms of population growth and movement, network sizes, and alternative patterns of life‐history strategy. It can also include carryover effects and density dependence and can model interspecific interactions and environmental perturbations. Our modeling approach bridges the gap between metapopulations and migratory populations by building upon previous work of Esler (2000), who posited that metapopulation theory can be applied to migratory birds during distinct seasons if the subpopulations are independent, and Taylor and Hall (2012), who developed a model explicitly linking a metapopulation model (Levins, 1970) to migratory species.

The flexibility of our framework stems not only from its ability to caricature different types of populations but also because it can accommodate varying degrees of model complexity. In the simplest scenario, functions *s*_*ij,t*_ *, p*_*ij,t*_, and *f*_*i,t*_ are constant. Covariates can be added to any of these functions, for example, *N*_*i*_ *,*_*t*_ could be included to model density‐dependent demographics. Furthermore, stochasticity can be easily incorporated in the model through simulation where parameter values are recursively sampled from user‐defined probability distributions. Last, our framework could be used as a common basis for fitting integrated population models to empirical data and parameter estimation using Bayesian hierarchical analysis (Schaub & Abadi, 2011).

Our common modeling framework is also useful for comparing impacts of environmental perturbations among sympatric populations. As an example, when the *Anaxyrus americanus* (American toad) and *Lithobates catesbeianus* (bullfrog) coexist in a landscape, the population‐level effects of anthropogenic stressors (e.g., mercury) on amphibian dynamics can be studied under one modeling framework (Willson & Hopkins, 2013; Willson, Hopkins, Bergeron, & Todd, 2012). Another benefit of using a consistent population modeling approach is to study interactions among different populations. Predation by *Ursus arctos* (grizzly bears) and *Canis lupus* (wolves) on *Cervus elaphus* (Greater Yellowstone elk) (Middleton et al., 2013) could be examined under our framework by modeling the three interacting networks. Model functions in such interacting networks can depend on the population size and parameters of the interacting species. For instance, the nodal update function for elk may depend not only on the elk population but also on the population size of wolves and bears at the node. This example also illustrates that metapopulations (bears and wolves) and migratory populations (elk) can be modeled under one unifying framework. In this way, a number of different types of species interactions, including competition and mutualisms, could be modeled using our framework, allowing for increased understanding of spatially structured community dynamics as well.

Our modeling framework provides the opportunity to improve understanding of movement ecology by unraveling the underlining processes shaping spatiotemporal population dynamics. A common demographic framework makes it straightforward to incorporate individual variation in movement strategies that almost always occur within highly mobile species. This can enhance our understanding of fitness benefits of different movement strategies (e.g., residency vs. partial vs. complete migration) and how such fitness variation influences the evolution of different movement strategies (McPeek & Holt, 1992; Morris, Diffendorfer, & Lundberg, 2004; Taylor & Norris, 2007).

Improved understanding of movement processes can in turn inform many potential management applications. It can be used, for example, to model the impact of habitat loss and changes in migratory flow or habitat quality along with other types of perturbations on population size, species persistence, distribution, and movement patterns including migration. The network model can also be used to quantify the per capita contribution of individual edges and nodes to population dynamics (Runge, Runge, & Nichols, 2006). Network topology measures can be used in these analyses, for example, to examine the robustness of spatial structure to perturbations such as node removal (Fortuna, Gomez‐Rodriguez, & Bascompte, 2006). Such considerations will no doubt prove crucial to our ability to anticipate and mediate the rapid pace of habitat fragmentation worldwide. By working within a common framework, there is less chance that comparative analyses are colored by model details rather than general principles. Above all, we sincerely hope that by providing a robust template for spatially structured population modeling, we encourage further work in this rapidly evolving field.

## CONFLICT OF INTEREST

None declared.

## AUTHOR CONTRIBUTIONS

This article originated as part of a NIMBioS Working Group. All authors contributed to the conception of the manuscript and the development of the model. CS, PF, JAB, DRN, and JF formulated model equations. JAB and CS created computer code. DRN and CS drafted an initial manuscript. All authors contributed to reviewing and editing the manuscript.

## DATA ACCESSIBILITY

*R scripts:* Zenodo entry https://doi.org/10.5281/zenodo.237369.
