# Supplementary material for: A general modeling framework for describing spatially structured population dynamics
Source: Ecol Evol. 2017 Nov 30;8(1):493–508. doi: 10.1002/ece3.3685 (PMC5756893; doi:10.1002/ece3.3685)
Supplement: Supplementary file 1 [file ECE3-8-493-s001.docx]

**Appendix S1.** *Model equations in matrix form*

Let the nodal population size after movement be given by the *n* × 1 vector ***N****_t_*, where *n* is the number of nodes in the network. Set ***P****_t_* as the stochastic matrix of edge transition probabilities and ***S****_t_* as the matrix of edge survival probabilities at time *t*. Both ***S****_t_* and ***P****_t_* are size *n* × *n*. The function for the nodal population size before movement (and after demographic updates at the node) is given by the *n* × 1 vector ***f****_t_*. Eqn 1 of the main text can be written in matrix form: ***N****_t+_*_1_ = (***S****_t_* _°_ ***P****_t_*)^T^***f****_t_*. Here ∘ represents the Hadarmard (entrywise) product and *T* indicates the transpose of a matrix. Note that ***S****_t_*, ***P****_t_,* and ***f****_t_* may be density-dependent, and consequently, the matrix equation may be nonlinear. Furthermore, we can write the total population size of the network at time *t* + 1, $N_{t+1}^{\mathrm{tot}}=\sum_{i=1}^{n} N_{i,t+1},$ in matrix form: $N_{t+1}^{\mathrm{tot}}\boldsymbol{=}\boldsymbol{1}^{\mathbf{T}}\left( \boldsymbol{S}_{t}\circ\boldsymbol{P}_{t} \right)^{T}\boldsymbol{f}_{t}$, where **1**^T^ is a 1 × *n* row vector of ones.
