# Supplementary material for: A general modeling framework for describing spatially structured population dynamics
Source: Ecol Evol. 2017 Nov 30;8(1):493–508. doi: 10.1002/ece3.3685 (PMC5756893; doi:10.1002/ece3.3685)

**Appendix S2.** *Species-specific examples*

***Ranunculus nodiflorus* (Metapopulation)**

The plant species, *Ranunculus nodiflorus* of the buttercup family, is a rare and endangered annual plant occuring in Spain, Portugal and France (Noel et. al. 2013). The plant grows only in ponds and reproduces by selfing (Kirchner et al. 2003) which produce seeds during April and May; plants die soon after reproduction (Noel et. al. 2013). Seeds float and are dispersed along water corridors that arise during flooding that connect adjacent ponds that operate as a typical metapopulation (Kirchner et al. 2003, Noel et al. 2006, Noel et. al. 2013). Some seeds germinate in autumn and others germinate in spring (Noel et al. 2006).

The model formulation for *Ranunculus nodiflorus* builds off of work done by two research groups. The network structure is based on a chain of ponds in Coquibus, France, as seen in the experimental study by Kirchner et al. (2003), in which plant dispersal through corridors connecting ponds is considered using a genetic analysis. Model parameters and density-dependent assumptions are based on the agent-based modeling approach of Noel et al. (2013). We simplify the dynamics by avoiding direct modeling of the seed bank. We use the general network equation (eqn 1 of the main text) and assume a density-dependent seed germination rate to match the carrying capacity enforced by Noel et al. (2013). An expansion of our model to include more specific seed bank modeling would only require the addition of age classes for the seeds and identification of appropriate parameters for these classes; for example, a decreasing germination and survival rate for older seeds. We avoided this addition because Noel et al. (2013) suggested that germination rates and annual survival of seeds in the seed bank were hard to study in the field and highly dependent on external conditions. Furthermore, the portion of seeds in the seed bank is estimated to be small (~0.2%).

***Network structure***

We model this metapopulation using a network of eight nodes based on pond maps from Kircher et al. (2003). We choose a representative chain of ponds from Figure 1(b) in their paper. The ponds were grouped into eight nodes as a linear network. Nodes have self-loops, for residents, and edges connecting to one or two neighboring nodes (Figure S1).

***Population classes***

The population is modeled using two age classes: seeds, denoted by a superscript *S*, and plants, denoted by a superscript *P*. The seed bank is not directly modeled. Survival rates and germination success of seeds in the seed bank was determined to be highly variable (Noel et al. 2013), making it difficult to model. Since only a small portion of seeds enter the seed bank (~0.2% Noel et al. 2013), we track only one seed class and assume that older seeds germinate and survive with the same probability as new seeds.

***Population dynamics***

*Time steps*

We track both classes through three times steps within an annual cycle: summer, autumn/winter, and spring. Each season is four months long. Summer (May-August) is when frutification and plant death occurs, autumn/winter (September-December) is when seeds disperse and few germinate to become plants, and spring (January-April) is when seeds disperse, most of the remaining seeds germinate, and flowering occurs. Seeds are allowed to disperse to adjacent nodes in every season with a high probability of remaining in their original node. In our simulation, we begin the annual cycle in the summer season, $t\in\left\{ 0,3,6,\ldots\right\}$, followed by winter, $t\in\left\{ 1, 4, 7,\ldots\right\},$and finally spring $t\in\left\{ 2, 5, 8,\ldots\right\}$.

*Model equations*

Let $N_{i,t}^{S}$ be the number of seeds and $N_{i,t}^{P}$ the number plants in node $i$at time $t$*.* The network model equations (eqn 1 of the main text) are given by:

$$N_{j,t+1}^{\bullet}=\sum_{i=1}^{n} s_{ij,t}^{\bullet}\cdot p_{ij,t}^{\bullet}\cdot f_{i,t}^{\bullet}$$

where the superscript indicates the stage class: $\bullet=\{S,P\}$. The formulations of $f_{i,t}^{\bullet}$, $p_{ij,t}^{\bullet}$,$s_{ij,t}^{\bullet}$, and parameters involved are described below for each class.

*Nodal update function*

Seeds: The nodal update function (eqn 2 of the main text) for seeds at each node $i$, $f_{i,t}^{S}\equiv f\left( N_{i,t}^{S}, N_{i,t}^{P}, \boldsymbol{\alpha}_{i,t} \right)$where the vector of node characteristics $\boldsymbol{\alpha}_{i,t}$ is described in the *Model parameters* subsection below, is given by

| $f_{i,t}^{S} =\underset{\begin{aligned} seeds that \\ survive \end{aligned}}{\underbrace{s_{i,t}^{S}\cdot N_{i,t}^{S}}}+ \underset{\begin{aligned} new seeds \\ from plants \end{aligned}}{\underbrace{R_{i,t}\cdot N_{i,t}^{P}}}-\underset{\begin{aligned} seeds that \\ transition \\ to plants \end{aligned}}{\underbrace{T_{i,t} \cdot s_{i,t}^{S}{\cdot N}_{i,t}^{S}}}$ | eqn S1 |
| --- | --- |

We assume that seed survival rates, $s_{i,t}^{S}$, are constant across seasons and the same for each node$.$ The factor $R_{i,t}$ in the second term of eqn S1 is the reproductive rate of plants producing seeds. Since reproduction only occurs in the summer, $R_{i,t}$ for all nodes $i$ is given by

$$R_{i,t}=\left\{ \begin{matrix} \begin{matrix} rs_{i,t}^{S}F \\ 0 \\ 0 \end{matrix} & \begin{matrix} t=0, 3, 6, \ldots\left( \mathrm{summer} \right) \\ t=1, 4, 7, \ldots(autumn/winter) \\ t=2, 5, 8, \ldots\left( \mathrm{spring} \right) \end{matrix} \end{matrix} \right.$$

where $r$ is the probability of a plant producing at least one fruit and $F$ is the number of seeds per fruit. The factor $T_{i,t}$ in the third term of eqn S1 represents the germination rate, or transition rate of seeds to plants. For all nodes $i,$seeds transition to plants in the autumn/winter and spring seasons:

| $T_{i,t}=\left\{ \begin{aligned} 0 &t=0, 3, 6, \ldots(summer) \\ 1-a-b &t=1, 4, 7, \ldots(autumn/winter) \\ \frac{b}{a+b} &t=2, 5, 8, \ldots(spring) \end{aligned} \right.$ | eqn S2 |
| --- | --- |

where $a$ is the proportion of seeds that do not germinate and enter the seed bank (not explicitly modeled, seeds remain in the node), and $b$ is the proportion of summer seeds that germinate in the spring.

Plants: The nodal update function for plants at each node $i$ and time step $t$, $f_{i,t}^{P}\equiv f\left( N_{i,t}^{S}, N_{i,t}^{P}, \boldsymbol{\alpha}_{i,t} \right)$, is given by

| $f_{i,t}^{P}=\underset{\begin{aligned} plants that \\ survive \end{aligned}}{\underbrace{s_{i,t}^{P}\cdot N_{i,t}^{P}}}+ \underset{\begin{aligned} seeds that successfully \\ transition to plants \end{aligned}}{\underbrace{\Psi_{i,t}\cdot T_{i,t}\cdot s_{i,t}^{S}{\cdot N}_{i,t}^{S}}}$ | eqn S3 |
| --- | --- |

We assume that plant survival rates, $s_{i,t}^{P}$, are the same for each node with none of the plants surviving the summer after they produce seeds and all plants surviving in the autumn/winter and spring seasons. The second term of eqn S3 is the product of seeds that survive, $s_{i,t}^{S}N_{i,t}^{S}$, the seed germination rate, $T_{i,t}$, given in eqn S2, and the factor $\Psi_{i,t}$, which is the density-dependent post-germination survival rate. Germination occurs in the autumn/winter and spring seasons with a post-germination survival rate depending on the number of existing plants, $N_{i,t}^{P}$, and the number of seeds that germinate in that season, $T_{i,t}s_{i,t}^{S}N_{i,t}^{S}$:

$$\Psi_{i,t}=\psi_{i,t}\exp\left( -\frac{N_{i,t}^{P}+T_{i,t}\cdot s_{i,t}^{S}{\cdot N}_{i,t}^{S}}{K_{i}} \right),$$

where $K_{i}$is the carrying capacity at node $i$ and the maximum germination rate is given by

$$\psi_{i,t}=\left\{ \begin{matrix} \begin{matrix} 0 \\ \gamma\\ \epsilon\end{matrix} & \begin{matrix} t=0, 3, 6, \ldots\left( \mathrm{summer} \right) \\ t=1, 4, 7, \ldots( autumn/winter) \\ t=2, 5, 8, \ldots\left( \mathrm{spring} \right) \end{matrix} \end{matrix} \right.$$

*Edge transition probabilities*

Seeds: Transition probabilities (eqn 3 of the main text) for dispersal are not dependent on the season, although this restriction could be lifted to better model seasonal flooding if data were available. We assume seeds remain at the node 90% of the time and only transition to an adjacent node 10% of the time. We further assume no preferential dispersion so the seeds have equal probability of going to any adjacent node (Figure S2).

Plants: Since plants do not move between nodes, then for every time step $t,$

$$p_{ij,t}^{P}=\left\{ \begin{aligned} 0 &i\neq j \\ 1 &i=j \end{aligned} \right.$$

*Edge survival probabilities*

Seeds: We assume a survival probability of 1 (eqn 4 of the main text) for every edge and at every time step.

Plants: Since plants do not move between nodes, then for every time step $t$,

$$s_{ij,t}^{P}=\left\{ \begin{aligned} 0 &i\neq j \\ 1 &i=j \end{aligned} \right.$$

*Model parameters*

The vector of node characteristics is given by

$$\boldsymbol{\alpha}_{i,t}=\left( s_{i,t}^{S},s_{i,t}^{P},T_{i,t},R_{i,t},K_{i},\psi_{i,t} \right).$$

Edge characteristics consist only of the edge transition and survival probabilities, which are equivalent across seasons. Model parameter values are given in Sample et al. (2017).

***Model outcome***

The network *Ranunculus nodiflorus* model is programmed using R version 3.2.1 (Sample et al. 2017). We assumed an initial plant population of $\boldsymbol{N}_{0}^{P}=[10,0,10,0,0,10,10,10],$ where ponds that were found to contain the plant in an initial 1999 study, Kirchner et. al. (2003), were assigned a small initial population. Initial seed population was set to zero.

The model converged to a steady state solution after 9 years, or 27 time steps. We see the initially small population of plants disperse to all ponds in the network and sustain a small, but persistent population. Figure S3 shows the population at the beginning of each summer season. The small seed population is due to the large germination rate in the spring, reducing the total seed population at the start of summer. In Figure S4, each class' population is plotted over one annual cycle at steady state. Total steady state population counts at the beginning of each season in the annual cycle are given in Table S1.

***Anas acuta* (Complete Migration)**

*Anas acuta* (northern pintail) is an example of a population that performs seasonal complete migration. The northern pintail is widely distributed in wetland regions; it breeds in the northern areas of North America, Europe, and Asia and winters close to the equator. We translate the model in Mattsson et al. (2012) into our modeling framework; parameter values and model assumptions match those of the paper.

***Network structure***

We model this migratory population using a network of five nodes with a set of three breeding nodes and a set of two wintering nodes. The breeding nodes named *AK*, *PR*, and *NU* (enumerated as node 1, 2 and 3) are located in northern North America. The wintering nodes, *CA* and *GC* (nodes 4 and 5), are in the southern portion of the range. The *PR* node also serves as a stopover site during spring migration (Figure S5).

***Population classes***

The population is modeled using two classes, females (*F*) and males (*M*), with two age classes for each sex, juveniles (*J*) and adults (*A*). The main reason to group the individuals in four different categories is differential node and edge survival rates.

***Population dynamics***

*Time steps*

A portion of the population uses a stopover site during spring migration. Thus, we divide the annual cycle into three time steps: breeding/fall migration, winter/spring migration, and spring stopover. Time steps vary in length, depending on the season. Breeding/fall migration takes place between April 15 and January 15, which includes breeding and fall migration through the end of the hunting season. Winter/spring migration takes place between January 15 and March 15, which includes winter survival and initial spring migration. Spring stopover takes place between March 15 and April 15, this includes initial survival at breeding nodes and a density dependent second spring migration to the final breeding locations.

In the breeding/fall time step, individuals survive and breed in *AK*, *PR*, and *NU* and then migrate to *CA* and *GC*. If hunting is included in the model, it happens during fall migration. During the winter/spring time step, individuals survive the winter in *CA* and *GC* and then migrate to *AK* and *PR*. During the spring stopover time step, some individuals will complete their migration by continuing on to *AK* or *NU* from *PR*. The rest of the population will remain as residents in nodes *AK* and *PR*. Our simulation begins at the start of the breeding/fall migration season, $t\in\left\{ 0,3,6,\ldots\right\}$, followed by winter/spring migration, $t\in\left\{ 1, 4, 7,\ldots\right\},$and finally spring stopover $t\in\left\{ 2, 5, 8,\ldots\right\}$.

*Model equations*

Let $N_{i,t}^{A\bullet}$ be the number of adult males or females and $N_{i,t}^{J\bullet}$ be the number of juvenile males or females in node $i$ at time $t$, where the superscript indicates the sex class: $\bullet=\{M,F\}$. Let the elements of vector $\boldsymbol{N}_{i,t}=(N_{i,t}^{AF}$ ,$N_{i,t}^{AM}$ ,$N_{i,t}^{JF}$ ,$N_{i,t}^{JM}$ ) consist of the population sizes for the four different classes at node $i$ and time $t$. The model equations (eqn 1 of the main text) become:

$$N_{j,t+1}^{A\bullet}=\sum_{i=1}^{n} s_{ij,t}^{A\bullet}\cdot p_{ij,t}^{A\bullet}\cdot f_{i,t}^{A\bullet},$$

$$N_{j,t+1}^{J\bullet}=\sum_{i=1}^{n} s_{ij,t}^{J\bullet}\cdot p_{ij,t}^{J\bullet}\cdot f_{i,t}^{J\bullet}.$$

The total number of males is expressed as the sum of adults and juveniles, $N_{i,t}^{M}=N_{i,t}^{AM}+N_{i,t}^{JM}.$ Likewise, the total number of females is $N_{i,t}^{F}=N_{i,t}^{AF}+N_{i,t}^{JF}$. These quantities will be involved in terms with density dependence.

*Nodal update function*

Adults: The function (eqn 2 of the main text) that represents adult survival and transitions into this group for each node $i$ at time step $t$, $f_{i,t}^{A\bullet}\equiv f\left( \boldsymbol{N}_{i,t}, \boldsymbol{\alpha}_{i,t} \right)$where the vector of node characteristics $\boldsymbol{\alpha}_{i,t}$ is defined in the *Model parameters* subsection below, is given by

$$f_{i,t}^{A\bullet}=\underset{\begin{aligned} adults that \\ survive \end{aligned}}{\underbrace{s_{i,t}^{A\bullet}{\cdot N}_{i,t}^{A\bullet}}}+\underset{\begin{aligned} juveniles that \\ transition to \\ adults and survive \end{aligned}}{\underbrace{T_{i,t}\cdot s_{i,t}^{A\bullet}{\cdot N}_{i,t}^{J\bullet}.}}$$

Juveniles transition to adults only in the winter/spring season (just after fall migration) so that

$$T_{i,t}=\left\{ \begin{matrix} 0 & t=0, 3, 6\ldots(breeding/fall) \\ 1 & t=1, 4, 7\ldots(winter/spring) \\ 0 & t=2, 5,8\ldots\left( \mathrm{stopover} \right) \end{matrix} \right.$$

Adults have constant node-specific survival during the breeding/fall season and stopover step. In the winter (i.e. post-hunting), it depends on density and other node characteristics:

| $s_{i,t}^{A\bullet}=\left\{ \begin{matrix} s_{i,t}^{\bullet} & t=0, 3, 6\ldots(breeding/fall) \\ s_{i\min}^{\bullet}+\frac{s_{i\max}^{\bullet}-s_{i\min}^{\bullet}}{1+e^{-Z_{t}}} & t=1, 4, 7\ldots(winter/spring) \\ s_{i,t}^{\bullet} & t=2, 5,8\ldots\left( \mathrm{stopover} \right) \end{matrix} \right.$ | eqn S4 |
| --- | --- |

where

$$Z_{t} = b_{i0}+b_{i1}(N_{i,t}^{F}+ N_{i,t}^{M}).$$

Juveniles: The nodal update function for juveniles, $f_{i,t}^{J\bullet}\equiv f\left( \boldsymbol{N}_{i,t}, \boldsymbol{\alpha}_{i,t} \right),$ exclusively represents the addition of offspring in node $i$ at time $t$,

$$f_{i,t}^{J\bullet}=\underset{\begin{aligned} juveniles born to \\ adult females \end{aligned}}{\underbrace{R_{i,t}{{\cdot s}_{i,t}^{AF}\cdot N}_{i,t}^{AF}}}$$

where $s_{i,t}^{AF}$ is given in eqn S4. Reproduction only occurs during the breeding/fall season:

| $R_{i,t}=\left\{ \begin{matrix} \exp\left[ a_{i0}+a_{i1}\left( N_{i,t}^{F}+N_{i,t}^{M} \right)+a_{i2}P_{i} \right] & t=0, 3, 6, \ldots(breeding/fall) \\ 0 & t=1, 4, 7\ldots(winter/spring) \\ 0 & t=2, 5,8\ldots\left( \mathrm{stopover} \right) \end{matrix} \right.$ | eqn S5 |
| --- | --- |

where $P_{i}$ is the number of ponds in node $i$, and $a_{ik}$ , for $k\in\{0,1,2\}$, are parameters specific to node *i.*  Note that eqn S5 implies that $N_{i,t}^{J\bullet}=0$ for $t=\{0, 3, 6, \ldots\}$ and $t=\{2, 5, 8, \ldots\}$. That is, all individuals are adults at the beginning of the breeding/fall season and at the beginning of the spring stopover step.

*Edge transition probabilities*

Edge transition probabilities (eqn 3 of the main text) do not depend on the sex or age class, $p_{ij,t}^{A\bullet}=p_{ij,t}^{J\bullet}\equiv p\left( \boldsymbol{N}_{i,t},\boldsymbol{\alpha}_{i,t}, \boldsymbol{\beta}_{ij,t} \right).$ Edge proportions are constant during the breeding/fall and winter/spring time steps. During the spring stopover step, edge proportions are constant except for node $i=2$ (node *PR*). In this case,

$$p_{2j,t}^{A\bullet}=p_{2j,t}^{J\bullet}=p_{2j,t} =\left\{ \begin{aligned} 1-\frac{\psi_{2}^{\max}}{1+e^{-Y_{t}}}, j=2 \\ \frac{\psi_{2j}\cdot\psi_{2}^{\max}}{1+e^{-Y_{t}}}, &j\neq2 \end{aligned} \right.$$

where

$$Y_{t}=\delta_{20}+\delta_{21}\left( N_{2,t}^{F}+N_{2,t}^{M} \right)+\delta_{22}P_{2.}$$

Figure S6 displays the transition probabilities for each of the three seasons.

*Edge survival probabilities*

Adults*:* Edge survival probabilities (eqn 4 of the main text), $s_{ij,t}^{A\bullet}\equiv s\left( \boldsymbol{\beta}_{ij,t} \right),$ are the same for adult males and females except during fall migration when hunting takes place,

$$s_{ij,t}^{A\bullet} =\left\{ \begin{matrix} s_{ij,t}^{A}\left( 1-\kappa_{ij,t}^{A\bullet} \right) & t=0, 3, 6, \ldots(breeding/fall) \\ s_{ij,t}^{A} & t=1, 4, 7\ldots\left( winter/spring \right) \\ s_{ij,t}^{A} & t=2, 5,8\ldots\left( \mathrm{stopover} \right) \end{matrix} \right.$$

where $\kappa_{ij,t}^{A\bullet}$ is a hunting mortality parameter, specific to the edge $ij$ and sex of the individual.

Juveniles: Edge survival probabilities are the same for male and female juveniles,$s_{ij,t}^{J\bullet}\equiv s\left( \boldsymbol{\beta}_{ij,t} \right),$ except during fall migration when hunting takes place,

$$s_{ij,t}^{J\bullet} =\left\{ \begin{matrix} s_{ij,t}^{J}\left( 1-\kappa_{ij,t}^{J\bullet} \right) & t=0, 3, 6, \ldots(breeding/fall) \\ 0 & t=1, 4, 7\ldots\left( winter/spring \right) \\ 0 & t=2, 5,8\ldots\left( \mathrm{stopover} \right) \end{matrix} \right.$$

where $\kappa_{ij,t}^{J\bullet}$ is a hunting mortality parameter for juveniles. Note that there are no juveniles at the end of winter/spring and stopover steps because all have transitioned to adults. Therefore, juvenile survival along the edges is not applicable, so we assign these values to be zero.

*Model parameters*

The vectors of node characteristics is given by

$$\boldsymbol{\alpha}_{i,t}\boldsymbol{=}\left( s_{i,t}^{F},s_{i,t}^{M},{P_{i},a_{ik},\delta_{ik},\psi_{i}^{\max},b_{i0}, b_{i1},s}_{i\min}^{F}, s_{i\max}^{F}, s_{i\min}^{M}, s_{i\max}^{M} \right)$$

for $k\in\left\{ 0,1,2 \right\}.$ The vector of edge characteristics is given by

$$\boldsymbol{\beta}_{ij,t}\boldsymbol{=}\left( s_{ij,t}^{A},s_{ij,t}^{J},\kappa_{ij,t}^{AM}, \kappa_{ij,t}^{AF}, \kappa_{ij,t}^{JM},\kappa_{ij,t}^{JF},\psi_{ij} \right)$$

Model parameter values are given in Sample et al. (2017).

***Model outcome***

The network Pintail model is programmed using R version 3.2.1 (Sample et al. 2017). We assumed an initial population of $\boldsymbol{N}_{0}^{AF}=\boldsymbol{N}_{0}^{AM}=[465000, 986850, 160650, 0, 0]$ for both the male and female adult populations and zero population for juveniles. This matches the initial population used in Mattsson et al. (2012).

The model converged to a steady state solution after 66 years, or 198 time steps. Figure S7 shows the population at the beginning of the summer season, before breeding. Figure S8 displays each class' population over one annual cycle at steady state. Steady state population sizes at the beginning of each season in the annual cycle are given in Table S2. We find a breeding population of 5.98 million, compared to 5.94 million found by Mattsson et al. (2012) in the absence of harvest.

***Cervus canadensis* (Partial Migration)**

*Cervus canadensis* (elk) are large herbivorous mammals occuring across North America. The best studied populations occur in, and adjacent to, Yellowstone National Park where the abundance of elk and those of their major predators, *Canus lupus* (wolves), have been monitored for decades (Middleton et al. 2013). Elk near Cody, Wyoming comprise a partial seasonally migratory population where one group of elk remain resident year-round in areas east of Yellowstone National Park and another group migrates seasonally from a shared overwintering grounds to breeding grounds in Yellowstone National Park (Middleton et al. 2013). Elk breed in polygynous groups of one or few males breeding with multiple females so generally females outnumber males approximately 4:1.

The network structure for the elk model is based on Global Positioning System (GPS) locations from a sample of migratory and resident elk in the Greater Yellowstone Ecosystem, USA by Middleton et al. (2013). Model parameters and density dependent assumptions are based on Middleton et al. (2013), Taper (2002) and Singer et al. (1997).

***Network Structure***

The elk population is modeled with a network of three nodes. Node 1 is the high-elevation summer range inside Yellowstone National Park, node 2 is the region where some migratory elk winter east of Yellowstone National Park, and node 3 is the foothill region northwest of Cody, Wyoming where some elk remain year-round and where a portion of migratory elk winter (Middleton et al 2013). The network structure is shown in Figure S9.

***Population classes***

The female population is modeled with two age classes, juveniles (*J*) and adults (*A*). Juveniles are elk less than 1 years old. Furthermore, we assume the ratio of adult females to adult males is 4:1 (Mack & Singer 1993) and the ratio of female juveniles to male juveniles is 1.5:1 (Houston 1982). The male population is not represented in our model. We assume there are enough males to allow for mating and number of males can be estimated based on the sex ratios specified above.

***Population dynamics***

*Time steps*

We divide the annual cycle into two time steps: winter/spring and summer/fall. Both seasons are about 6-months long. In the winter/spring time step, all individuals in node 2 and migratory elk wintering in node 3 will migrate to node 1 for the summer whereas resident elk in node 3 will remain in that node. In the summer/fall time step, all elk in node 1 will migrate to either node 2 or node 3. Resident elk in node 3 will remain there in the next time step. Our simulation begins at the start of winter/spring, $t\in\left\{ 0,2,4,\ldots\right\}$, followed by summer/fall, $t\in\left\{ 1, 3, 5,\ldots\right\}$.

*Model equations*

The network model equations (eqn 1 of the main text) is given by

$$N_{j,t+1}^{\bullet}=\sum_{i=1}^{n} s_{ij,t}^{\bullet}\cdot p_{ij,t}^{\bullet}\cdot f_{i,t}^{\bullet}$$

where the superscript indicates the stage class: $\bullet=\{J,A\}$.

*Nodal update function*

Adults: The function in eqn 2 of the main text, $f_{i,t}^{A}\equiv f\left( N_{i,t}^{A},N_{i,t}^{J},\boldsymbol{\alpha}_{i,t} \right)$ where $\boldsymbol{\alpha}_{i,t}$ is described in the *Model parameters* subsection below, represents adult survival and transitions into the adult group for node $i$ at time $t$ and is given by:

$$f_{i,t}^{A}=\underset{\begin{aligned} adults that \\ survive \end{aligned}}{\underbrace{s_{i,t}^{A}{\cdot N}_{i,t}^{A}}}+\underset{\begin{aligned} surviving juveniles \\ that transition \\ to adults \end{aligned}}{\underbrace{T_{t}\cdot s_{i,t}^{J}\cdot N_{i,t}^{J}}}$$

We assume female juveniles transition to adults every summer, therefore,

| $T_{t}=\left\{ \begin{matrix} 0 & t=0, 2, 4, \ldots(winter/spring) \\ 1 & t=1, 3, 5, \ldots(summer/fall) \end{matrix} \right.$ | eqn S6 |
| --- | --- |

We also assume that annual adult survival is density-dependent. A previous model by Taper (2002) proposed the expression$\exp\left( -0.219\cdot\left( \frac{N}{K} \right)^{3.77} \right)$ for annual survival. Since we assume seasons are about 6-months long, we set the seasonal adult survival rate for all nodes and both seasons as

| $s_{i,t}^{A}=\sqrt{\exp\left( -0.219\cdot\left( \frac{N_{i,t}^{A}+N_{i,t}^{J}}{K_{i}^{A}+K_{i}^{J}} \right)^{3.77} \right)}.$ | eqn S7 |
| --- | --- |

The carrying capacity of node$i$ for adult and juvenile females is $K_{i}^{A}$ and $K_{i}^{J}$, respectively. Juvenile survival, $s_{i,t}^{J},$ is constant during the winter/spring season and density-dependent in the summer/fall season (Singer et al. 1997):

| $s_{i,t}^{J}=\left\{ \begin{matrix} s_{t}^{0} & t=0, 2, 4, \ldots(winter/spring) \\ s_{t}^{0}\exp\left( 1-\frac{N_{i,t}^{A}+N_{i,t}^{J}}{K_{i}^{A}+K_{i}^{J}} \right) & t=1, 3, 5, \ldots(summer/fall) \end{matrix} \right.$ | eqn S8 |
| --- | --- |

Juveniles: The function, $f_{i,t}^{J}\equiv f\left( N_{i,t}^{A},N_{i,t}^{J},\boldsymbol{\alpha}_{i,t} \right),$ which represents juveniles’ survival, recruitment, and transitions out of this group, is given by

| $f_{i,t}^{J}=\underset{\begin{aligned} surviving juveniles \\ that do not transition \\ to adults \end{aligned}}{\underbrace{(1-T_{t})\cdot s_{i,t}^{J}\cdot N_{i,t}^{J}}}+\underset{female calves born}{\underbrace{0.6\cdot r_{i,t}\cdot s_{i, t}^{A}\cdot N_{i, t}^{A}}}$ | eqn S9 |
| --- | --- |

where $T_{t}$, $s_{i,t}^{J},$ and $s_{i,t}^{A}$ are given in eqns S6, S7 and S8. Reproduction rates, $r_{i,t},$ are estimated by the proportion of pregnant elk, using a weighted average for all age classes presented in Fig. 3 of Middleton et al. (2013), and assumes that cows do not have twins. Reproduction rate is higher for residents (0.86) than for the migratory (0.68) subpopulation and it only occurs during the summer/fall time step:

$$r_{i,t}=\left\{ \begin{matrix} 0 & t=0, 2, 4, \ldots(winter/spring) \\ \mathrm{constant} & t=1, 3, 5, \ldots(summer/fall) \end{matrix} \right.$$

The factor 0.6 in the second term of eqn S9 represents the proportion of calves that are female.

*Edge transition probabilities*

Edge transition probabilities, $p_{ij,t}^{\bullet}\equiv p\left( N_{i,t}^{\bullet},\boldsymbol{\alpha}_{i,t}, \boldsymbol{\beta}_{ij,t} \right),$ for juveniles and adults are constant and the same during fall migration, but density-dependent and different during spring migration. This density-dependence accounts for the inheritance of a movement pathway. An individual inherits its movement path because a resident elk will remain a resident and a migratory elk will remain migratory. For the winter/spring season, $t=\{0, 2, 4, \ldots$}, all individuals in node 2 migrate to node 1, $p_{21,t}^{\bullet}=1.$ The only other non-zero edge transition probabilities (from node $3$ to nodes $1$ and 2) are density-dependent:

$$p_{31,t}^{\bullet}=1-\frac{M_{33,t-1}^{\bullet}}{N_{3,t}^{\bullet}},$$

$$p_{33,t}^{\bullet}=\frac{M_{33,t-1}^{\bullet}}{N_{3,t}^{\bullet}}.$$

Note that $M_{33,t-1}^{\bullet}$ is given in eqn 4 of the main text and represents the number of individuals (of the specified class) that were residents in node 3 in the previous time step.

For the summer/fall season, $t=\{1,3,5, \ldots\}$, the resident elk population in node 3 will remain there, $p_{33,t}^{\bullet}=1.$ The other non-zero edge transition probabilities account for the migrants traveling from node 1 to nodes 2 and 3, $p_{12,t}^{\bullet}$ and $p_{13,t}^{\bullet}$ and are given in Sample et al. (2017). Transition probabilities for each season are given in Figure S10.

*Edge survival probabilities*

We assume all individuals survive movement: $s_{ij,t}^{\bullet}=1$ for all $t$ and all $i,j\in\{1,\ldots,n\}$.

*Model parameters*

To determine values for carrying capacity, we assume it to be proportional to area. Nodes 2 and 3 have an area of 718 km^2^ and 1093 km^2^, respectively (Middleton et al 2013). Given a total of 3600 adult females in the winter (Middleton et al 2013), we set $K_{2}^{A}=1427$and $K_{3}^{A}=2173.$ Furthermore, we estimate the carrying capacity of node 1 as $K_{1}^{A}=\frac{K_{2}^{A}}{p_{12,f}}=1640$, where $p_{12,f}$denotes the proportion of individuals that migrate from node 1 to node 2 in the fall (~0.87). To determine the carrying capacity for juveniles, we assume a female calf:cow ratio of 0.15 and 0.24 in nodes 2 and 3, respectively. Thus, $K_{2}^{J}=214$ and $K_{3,w}^{J}=522.$

The vector of node characteristics is given by

$$\boldsymbol{\alpha}_{i,t}\boldsymbol{=}\left( s_{t}^{0}, r_{i,t},K_{i}^{A},K_{i}^{J} \right).$$

Model parameter values are given in Sample et al. (2017).

***Model outcome***

The network Elk model is programmed using R version 3.2.1 (Sample et al. 2017). We assumed an initial population at the start of winter of $\boldsymbol{N}_{0}^{A}=[0, 1427, 2173]$ for adults and zero calves.

The model converged to a steady state solution after 16 years, or 32 time steps. Figure S11 shows the population at the beginning of each summer season, before breeding. Figure S12 displays each class' population over one annual cycle at steady state. Total steady state population counts at the beginning of each season for an annual cycle are given in Table S3. Under this model parameterization, the female adult population in the beginning of winter was determined to be 3500, which is comparable to the 3600 estimated by Middleton et al. (2013).

The calf:cow ratio before breeding in the beginning of summer is 0.31 for the migratory subpopulation and 0.37 for the resident subpopulation. The calf:cow ratio at the beginning of winter is 0.36 in node 2 (migratory subpopulation) and 0.40 in node 3 (migratory and residents). These results align with observations from the mid-1990s shown in Fig. 2 of Middleton et al. (2013).

***Danaus plexippus* (Stepping-stone Migration)**

Each autumn, *Danaus plexippus* (monarch butterflies) in eastern North America migrate from breeding areas in the northern U.S. and southern Canada to non-breeding areas in central Mexico. At the end of the six-month non-breeding season, monarchs begin to mate and migrate north in March to breeding grounds in the southern U.S. Remigrating butterflies lay eggs then die whereupon their eggs develop into caterpillars and then butterflies which continue to fly north and recolonize the entire breeding distribution in successive breeding generations until September. The last generation of monarchs eclose in a non-reproductive state (diapause) and migrate south en masse to the overwintering colonies in Mexico. The recolonization over multiple breeding generations and return migration to the non-breeding grounds is represented as stepping-stone migration patterns. We convert the model presented in Flockhart et al (2015) to our network-based model.

***Network Structure***

The monarch population is modeled using a network of four nodes representing regions of eastern North America: Mexico (*M*), South (*S*), Central (*C*), and North (*N*), enumerated 1 through 4, respectively. Mexico is considered a wintering node and the other three nodes are breeding nodes: *S*, *C*, and *N* (Figure S13).

***Population classes***

In this model, we track only adult females through their main life history events and transitions.

***Population dynamics***

*Time steps*

An annual cycle consists of seven time steps: Winter, April, May, June, July, August, and September. In the Winter time step, the entire population resides at node *M* and then migrates to *S.* In April, the population either stays in node *S* or moves to node *C.* In May, the populations at nodes *S* and *C* move/reside in nodes *C* and *N.* The population then moves between nodes *C* and *N* in June and July. In August, monarchs will move to *S* and *C.* In September, all individuals migrate back to the wintering site *M*. We assume the monthly seasons April – September are of equal length and the Winter season is six months long.

*Model equations*

Let $N_{i,t}$ be the number of adult females in node $i$ at time $t$ so that our model equations (eqn 1 of the main text) are

$$N_{j,t+1}=\sum_{i=1}^{n} s_{ij,t}\cdot p_{ij,t}\cdot f_{i,t}$$

*Nodal update function*

The nodal update function of eqn 2 of the main text, $f_{i,t}\equiv f\left( N_{i,t},\boldsymbol{\alpha}_{i,t} \right)$where $\boldsymbol{\alpha}_{i,t}$ is the vector of node characteristics described below in the *Model parameters* subsection, accounts for survival and reproduction:

$$f_{i,t}\boldsymbol{=}\underset{\begin{aligned} adults that \\ survive \end{aligned}}{\underbrace{s_{i}^{A}\cdot N_{i,t}}}+\underset{\begin{aligned} eggs that survive and \\ transition to adults \end{aligned}}{\underbrace{s_{i}^{A}\cdot s_{i}^{P}\cdot s_{i}^{L}\cdot E\cdot N_{i,t}}}$$

Here, $E$ is the number of eggs per female per month, $s_{i}^{A}$ is adult survival and $s_{i}^{P}$ is pupal survival at node *i*. Larval survival is dependent on egg density per milkweed stem at node *i* and is given by

$$s_{i}^{L}=\frac{c(1+\exp\left( a \right))}{1+\exp\left( a+b\frac{E}{m_{i}}N_{i,t} \right)},$$

where $m_{i}$ is the number of milkweed in node *i*. The larval survival function is taken from Flockhart et al (2012), where $a=-1.0175, b=0.1972$ and $c=0.0327.$

*Edge transition probabilities*

Edge transition probabilities of eqn 3 of the main text, $p_{ij,t},$ vary across seasons but are assumed to be constant each year. Transition probabilities are derived from table S3 in Flockhart et al. (2015). These transition probabilities were calculated based on monarch butterflies that were captured by season across the breeding distribution. Using the capture location of a butterfly and an analysis of the stable isotope values in its wings, which can be used to assign the node with the highest probability of being the natal origin of the monarch, we tabulated the transition probabilities for each season. The node where a butterfly was captured was considered its destination node and the node of natal origin was considered its origin node. See Figure S14 for an illustration of edge transition probabilities for all seven seasons.

*Edge survival probabilities*

The edge survival probabilities, $s_{ij.t}$, given in eqn 4 of the main text are constant for a given time step $t$. Survival probabilities were derived from an expert elicitation exercise as presented in Flockhart et al. (2015).

*Model parameters*

To determine the number of eggs per female per month, $E$, we assume a lifetime egg output for butterflies to be 715 eggs (Oberhauser 1997). We scaled fecundity by 0.75 and assumed a 50:50 sex ratio to obtain $E=268$as the number of eggs per female per month$.$ The vector of node characteristics is given by

$$\boldsymbol{\alpha}_{i,t}\boldsymbol{=(}s_{i}^{A},s_{i}^{P},m_{i})$$

Edge characteristics consist only of the edge transition and survival probabilities. Model parameter values are given in Sample et al. (2017).

***Model outcome***

The network Monarch model is programmed using R version 3.2.1 (Sample et al. 2017). We assumed an initial population of $\boldsymbol{N}_{0}=[28250000, 0, 0]$. The model converged to a steady state solution after 4 years, or 32 time steps. Figure S15 shows the population at the beginning of each winter season. In Figure S16, the monarch population fluctuates over one annual cycle at steady state. Steady state population counts at the beginning of each season over an annual cycle are given in Table S4.

**References**

Flockhart, D.T.T., Martin, T.G., & Norris, D.R. (2012). Experimental examination of intraspecific density-dependent competition during the breeding period in monarch butterflies (*Danaus plexippus*). *PloS One*, 7(9), e45080.

Flockhart, D.T.T., Pichancourt, J.B., Norris, D.R. and Martin, T.G. (2015). Unravelling the annual cycle in a migratory animal: breeding-season habitat loss drives population declines of monarch butterflies. *Journal of Animal Ecology,* 84, 155–165.

Houston, D. G. (1982). *The northern Yellowstone elk: ecology and management.* Macmillan, New York, New York, USA.

Kircher, F., Ferdy, J.B., Andalo, C., Colas, B., & Moret, J. (2003). Roles of corridors in plant dispersal: an example with the endangered *Ranunculus nodiflorus*. *Conservation Biology,* 17, 401-410.

Mack, J. A., & Singer, F. J. (1993). *Population models for elk, mule deer, and moose on Yellowstone's northern range* (No. Scientific Monograph NPS/NRYELL/NRSM-93/22, pp. 270-305). National Park Service.

Mattsson, B. J., Runge, M. C., Devries, J. H., Boomer, G. S., Eadie, J. M., Haukos, D. A., et al. (2012). A modeling framework for integrated harvest and habitat management of North American waterfowl: Case-study of northern pintail metapopulation dynamics. *Ecological Modelling*, 225, 146-158.

Middleton, A. D., Kauffman, M. J., McWhirter, D. E., Cook, J. G., Cook, R. C., Nelson, A. A., et al. (2013). Animal migration amid shifting patterns of phenology and predation: lessons from a Yellowstone elk herd. *Ecology*, 94(6), 1245-1256.

Noël, F., Porcher, E., Moret, J., & Machon, N. (2006). Connectivity, habitat heterogeneity, and population persistence in *Ranunculus nodiflorus*, an endangered species in France. *New Phytologist*, 169(1), 71-84.

Noël, F., Machon, N., & Robert, A. (2013). Integrating demographic and genetic effects of connections on the viability of an endangered plant in a highly fragmented habitat. *Biological Conservation*, 158, 167-174.

Oberhauser, K. S. (1997). Fecundity, lifespan and egg mass in butterflies: effects of male‐derived nutrients and female size. *Functional Ecology*, 11(2), 166-175.

Sample, C., Fryxell, J., Bieri, J., Federico, P., Earl, J., Wiederholt, R., *et al*. (2017). *NIMBioS-NetworkCode/MigrationNetwork: Generalized Network Framework Code*. Zenodo. Available at: http://doi.org/10.5281/zenodo.237369.

Singer, F. J., Harting, A., Symonds, K. K., & Coughenour, M. B. (1997). Density dependence, compensation, and environmental effects on elk calf mortality in Yellowstone National Park. *The Journal of Wildlife Management*, 12-25.

Taper, M. L., & Gogan, P. J. (2002). The northern Yellowstone elk: density dependence and climatic conditions. *The Journal of Wildlife Management*, 106-122.

**Tables**

Table S1. Total plant and seed populations over one annual cycle at steady state.

| **Season** | **Seeds** | **Plants** |
| --- | --- | --- |
| Summer | 4 | 342 |
| Autumn/Winter | 3942 | 0 |
| Spring | 1189 | 75 |

Table S2. Total pintail populations over one annual cycle at steady state.

| **Season** | **Adult Females** | **Adult Males** | **Juvenile Females** | **Juvenile Males** |
| --- | --- | --- | --- | --- |
| Breeding/Fall | 2,332,055 | 3,651,902 | 0 | 0 |
| Winter/Spring | 1,696,110 | 3,213,489 | 985,778 | 985,778 |
| Spring Stopover | 2,332,055 | 3,651,902 | 0 | 0 |

Table S3. Total elk populations over one annual cycle at steady state.

| **Season** | **Female Calves** | **Female Adults** |
| --- | --- | --- |
| Winter/Spring | 1318 | 3507 |
| Summer/Fall | 949 | 2968 |

Table S4. Total monarch population over one annual cycle at steady state.

| **Season** | **Adult Females** |
| --- | --- |
| Winter | 104,369,878 |
| April | 50,678,510 |
| May | 65,711,517 |
| June | 86,725,288 |
| July | 134,481,626 |
| August | 142,239,303 |
| September | 128,489,061 |

**Figures**

Figure S1. The network structure of the plant example with eight nodes and all possible connections. The map of the chain of ponds was taken from Kircher et al. (2003).

Figure S2. Network structure for the 8-node metapopulation. Transition probabilities are given for seeds and plants. Since plants do not disperse, the network is disconnected and all plants remain in their node. The network structure and transition probabilities are the same for all three seasons.*
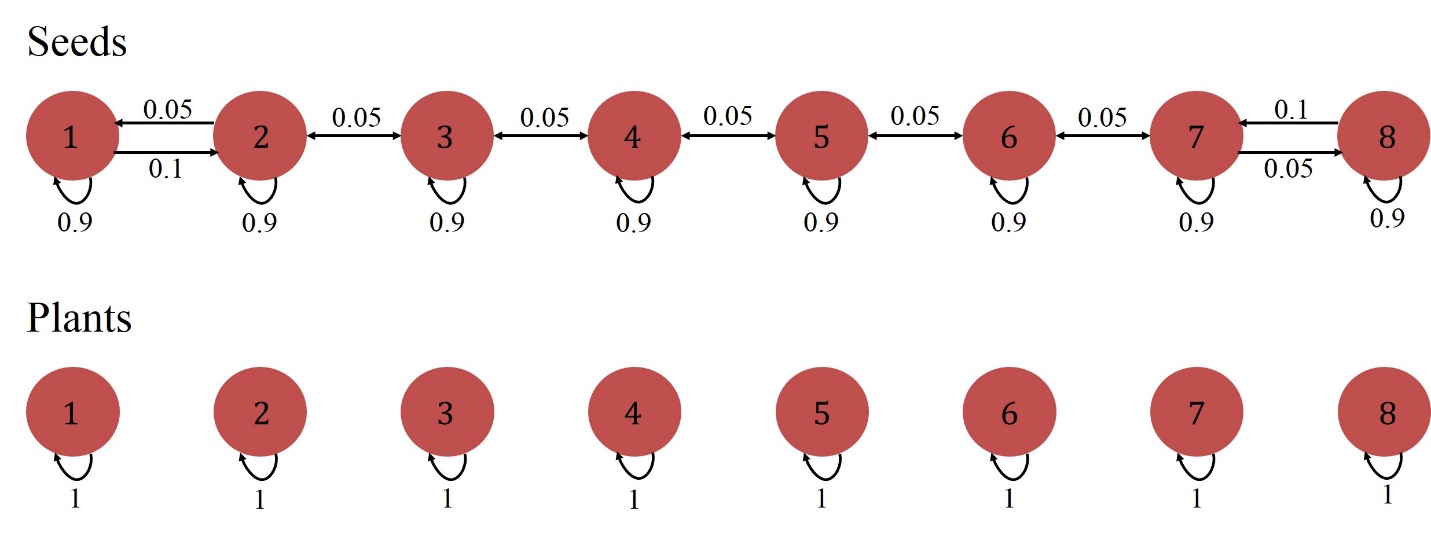
*

Figure S3: Seed and plant population at the beginning of summer, before fruiting.
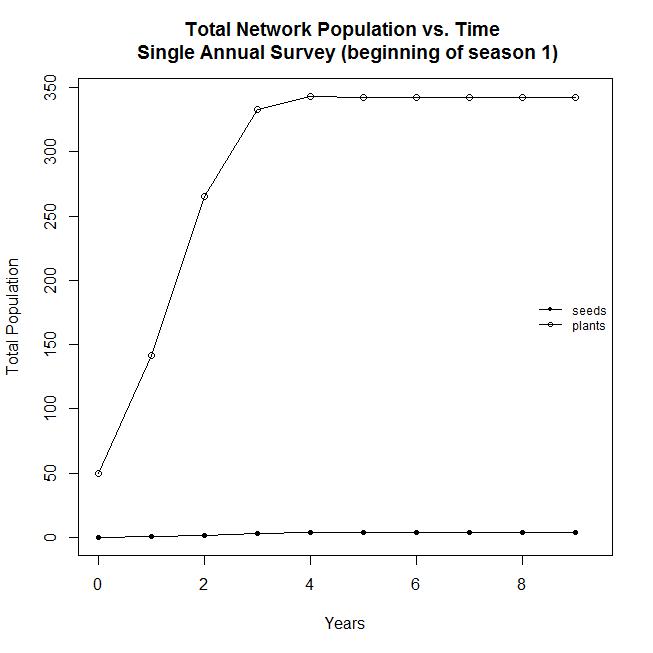


Figure S4: Plant and seed population over one annual cycle at steady state.


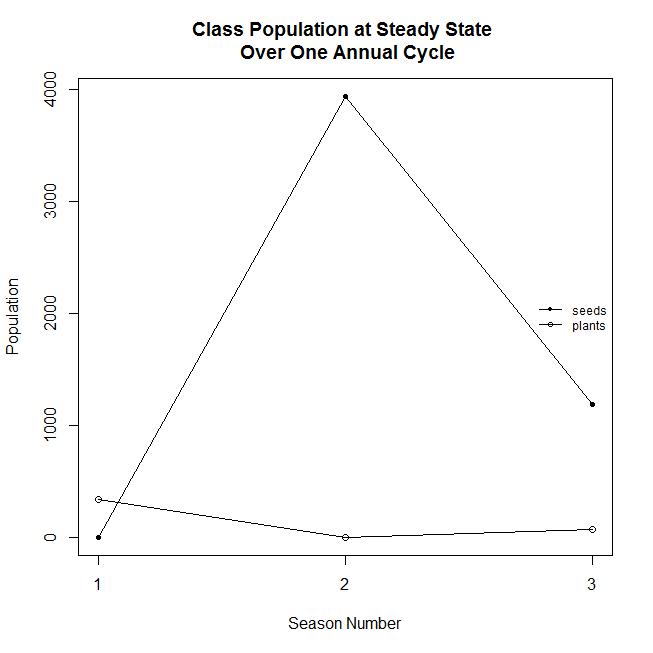


Figure S5. The network structure of the pintail example with five nodes and all possible connections. The map adapted from Mattsson et al. (2012).

Figure S6. Network structure with three breeding nodes and two nonbreeding nodes. This is an example of a seasonal complete migration. Edge transition probabilities are shown (same for all classes), where DD indicates a density-dependent transition probability.

*
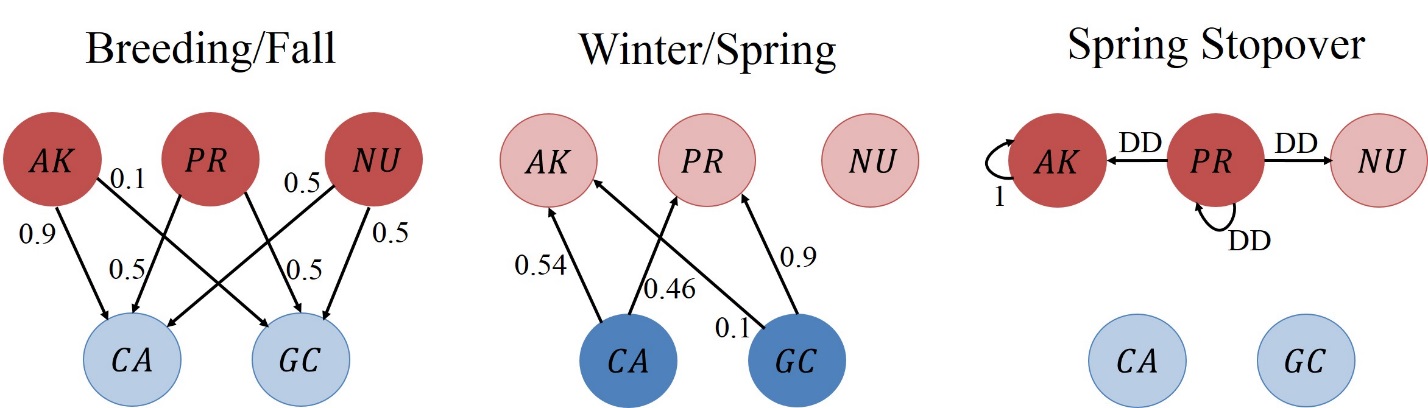
*

Figure S7. Pintail population at the beginning of summer, before breeding. The juvenile population is zero because we are counting the annual population before breeding occurs.


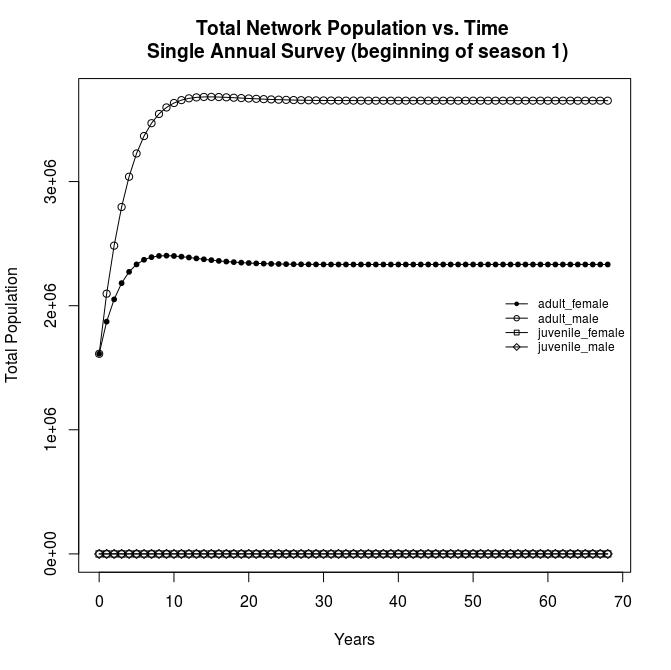


Figure S8. Pintail population over one annual cycle at steady state. Population sizes of juvenile males are females are equivalent.


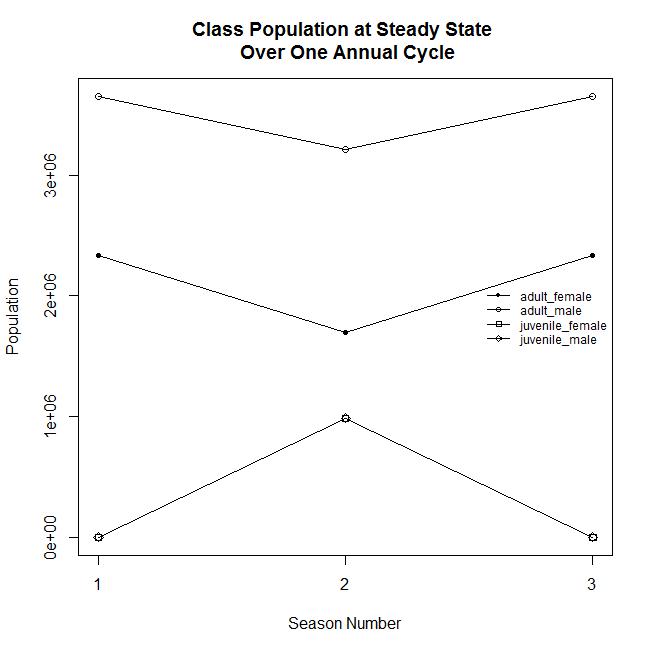


Figure S9. The network structure of the elk example with three nodes and all possible connections. The map was taken from Middleton et al. (2013).

Figure S10. Network structure for the three nodes and two seasons. This is an example of a seasonal partial migration. Transition probabilities are given. Density-dependent transition probabilities are indicated by DD, which is differ for adults and juveniles. The constant transition probabilities are the same for both classes.


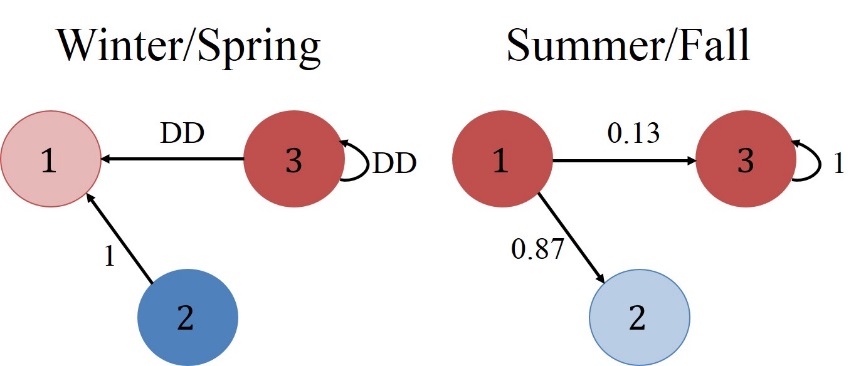


Figure S11. Female elk population at the beginning of summer, before breeding.


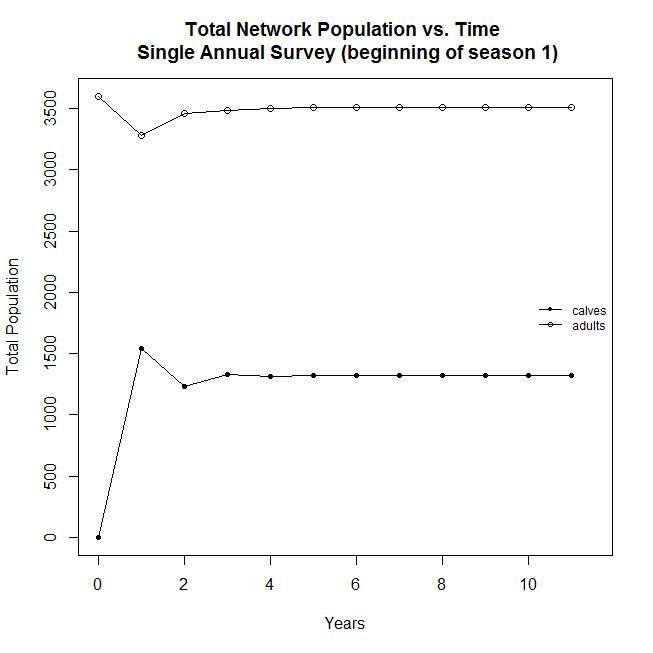


Figure S12. Female elk population over one annual cycle at steady state.


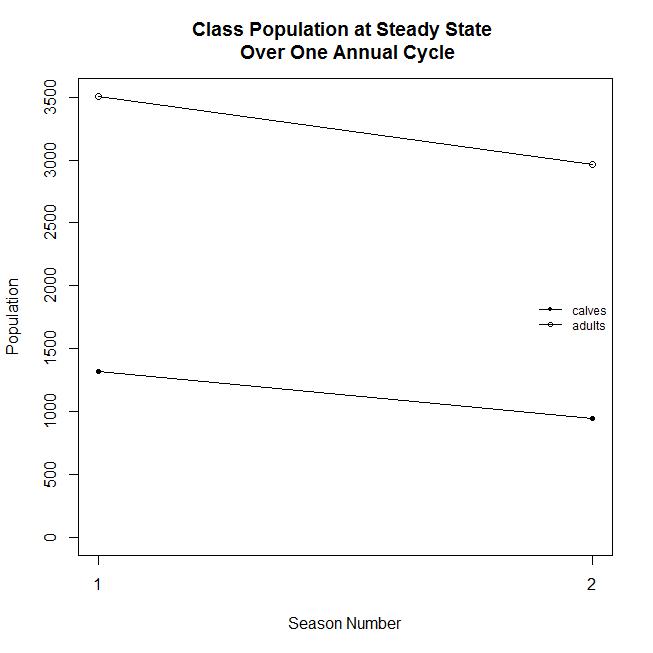


Figure S13. The network structure of the monarch example with three nodes and all possible connections. The map was taken from Flockhart et al. (2015).

Figure S14. Network structure for the four nodes and seven seasons. This is an example of stepping-stone migration. Transition probabilities are given.


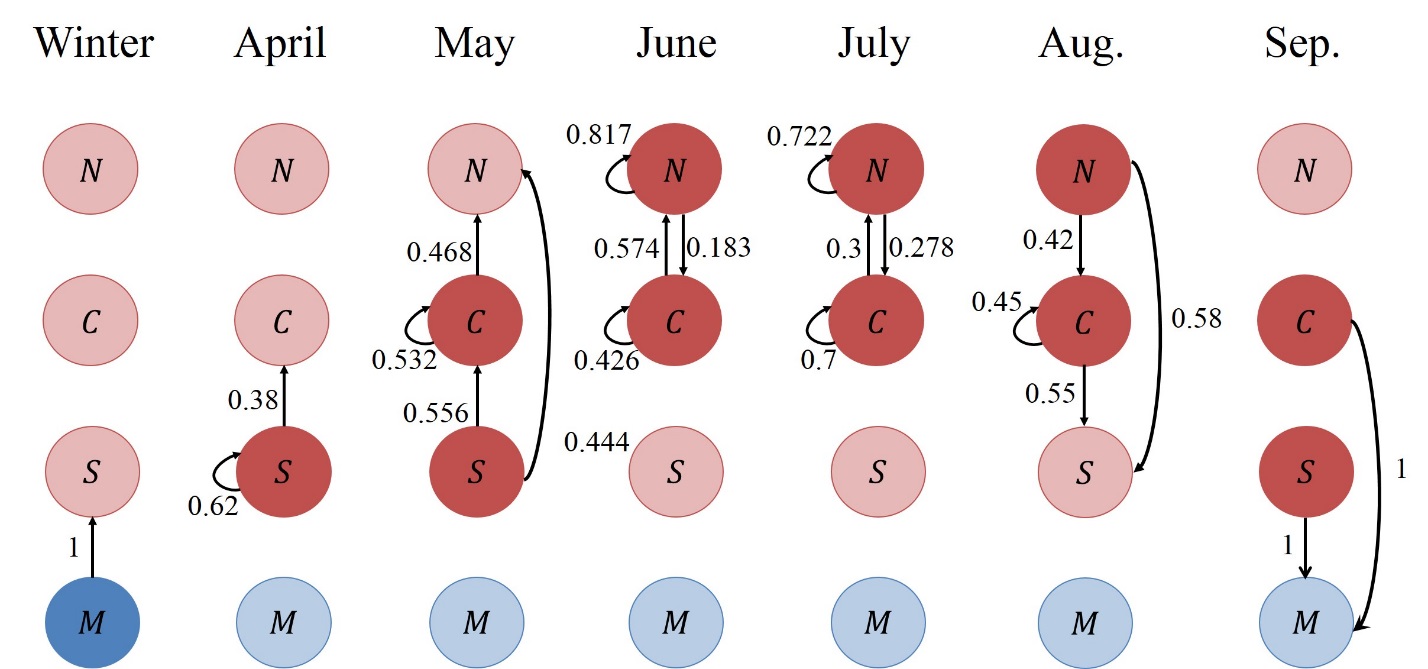


Figure S15. Adult female monarch population at the beginning of winter.


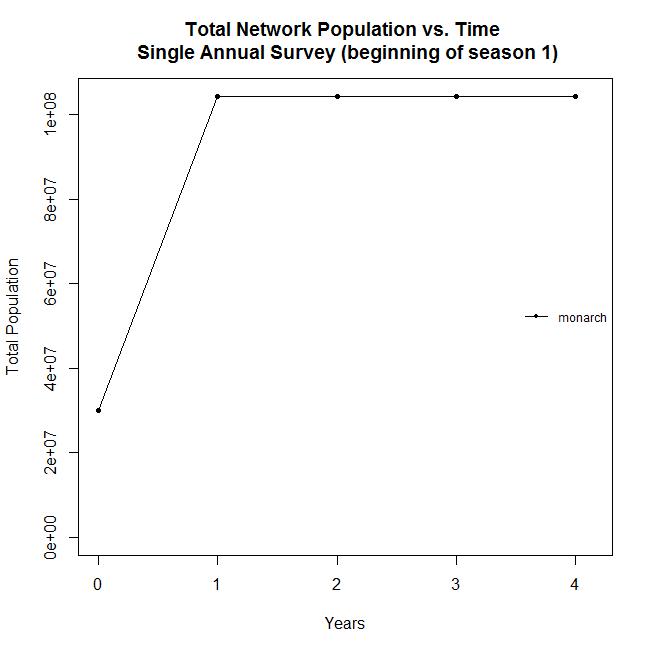


Figure S16. Adult female monarch population over one annual cycle at steady state.


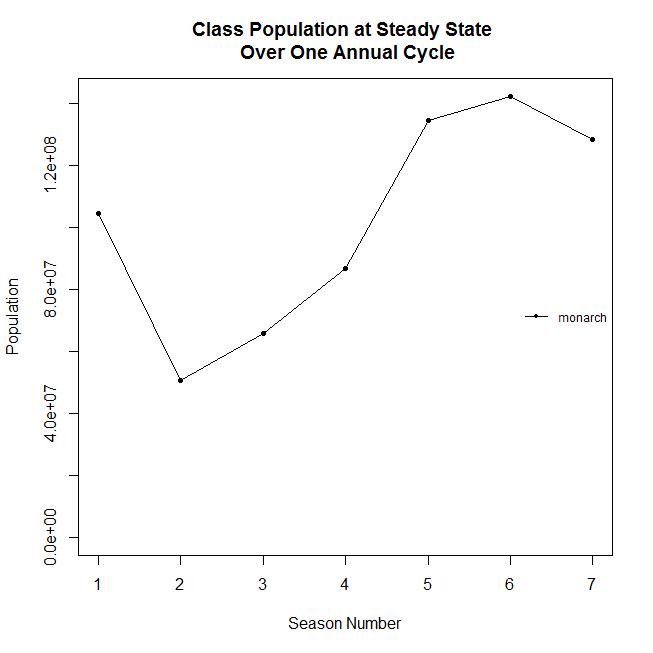

Supplement: Supplementary file 2 [file ECE3-8-493-s002.docx]
